# Cell Death in Skeletal Muscle Diseases: Diverse Roles and Pathological Processes

**DOI:** 10.3390/cells15090744

**Published:** 2026-04-22

**Authors:** Ya-Lan Yang, Liang Guo

**Affiliations:** 1 Key Laboratory of Exercise and Health Sciences of the Ministry of Education, Shanghai University of Sport, Shanghai 200438, China; 2421518040@sus.edu.cn; 2 School of Exercise and Health and Collaborative Innovation Center for Sports and Public Health, Shanghai University of Sport, Shanghai 200438, China; 3 Shanghai Key Lab of Human Performance, Shanghai University of Sport, Shanghai 200438, China; 4 Shanghai Frontiers Science Research Base of Exercise and Metabolic Health, Shanghai University of Sport, Shanghai 200438, China

**Keywords:** cell death, Duchenne muscular dystrophy, glycogen storage diseases, mitochondrial myopathies, idiopathic inflammatory myopathy, sarcopenia, rhabdomyolysis, myasthenia gravis

## Abstract

Skeletal muscle is vital for movement and metabolism, and its dysfunction underpins disorders like muscular dystrophy and sarcopenia, severely impacting life quality. In these diseases, various cell death pathways are pivotal, driving core pathological features such as fiber loss and chronic inflammation. This study reviews the central role of cell death in skeletal muscle diseases, and analyzes its roles and mechanisms in genetic muscle disorders such as Duchenne muscular dystrophy (DMD), glycogen storage diseases (GSD), mitochondrial myopathies, as well as acquired muscle disorders such as idiopathic inflammatory myopathy, sarcopenia, rhabdomyolysis, and myasthenia gravis (MG). We also explore the potential of cell death-related molecules as biomarkers and discuss emerging therapeutic strategies that target these pathways, aiming to provide new insights for diagnosis and treatment.

## 1. Introduction

Skeletal muscle, constituting over 40% of human body mass, serves not only as the primary effector of movement but also regulates glucose/lipid metabolism and systemic energy homeostasis [1]. By virtue of its contractile and metabolic functions, skeletal muscle supports daily activities and physiological demands, and its dysfunction affects hundreds of millions of people worldwide. Epidemiological data show that approximately one in every 3500 male infants will develop Duchenne muscular dystrophy (DMD), and these patients usually die from respiratory or heart failure before the age of 20 [2]. The overall incidence of glycogen storage disease (GSD) in the population is estimated to be 1 case per 20,000–43,000 people, belonging to the rare disease category [3]. Mitochondrial myopathy is also a rare disease, with a prevalence of about 1 in 5000 [4]. The annual incidence of inflammatory myopathies is between 0.2 and 2 cases per 100,000 individuals, and a large number of incidences are associated with malignant tumors, cardiovascular events, pulmonary diseases and infections [5]. The prevalence of sarcopenia among people over 60 years old is as high as 10% to 27%, and it is significantly associated with the risks of cardiovascular diseases and type 2 diabetes [6]. Rhabdomyolysis (RML), frequently induced by trauma or drug toxicity, occurs across all age groups and both sexes. Its precise incidence remains elusive due to limited prospective studies and underreporting of mild cases [7,8]. Worldwide, the prevalence rate of myasthenia gravis (MG) is approximately between 150 and 250 cases per million population [9]. This disorder drives progressive muscle atrophy and functional decline while precipitating systemic complications (e.g., renal failure, cardiomyopathy), representing a substantial public health burden.

In recent years, cell death has emerged as a critical biological process driving the initiation and progression of skeletal muscle disorders. Beyond causing myofiber loss, cell death extensively contributes to skeletal muscle pathology by modulating inflammation, metabolic homeostasis, and tissue regeneration. Historically, cell death was dichotomized into apoptosis and necrosis [10]. Advances in molecular biology and pathology have revealed novel regulated cell death modalities, such as pyroptosis, ferroptosis, and autophagic cell death. These newly characterized pathways exhibit distinct molecular signatures and signaling cascades, underscoring the pathophysiological complexity of skeletal muscle diseases. Classic apoptosis and necrosis have established roles in skeletal muscle dysfunction [11]. However, with the discovery of new types of death pathways such as necroptosis, autophagic, pyroptosis, ferroptosis, cuproptosis, disulfide death, and NETosis, the scope of research has been further expanded. For instance, pyroptosis amplifies inflammation through IL-1β/IL-18 release, driving immune cell infiltration in inflammatory myopathies [12,13]. Ferroptosis induces metabolic collapse in myofibers via iron-dependent lipid peroxidation, mechanistically linked to the oxidative stress microenvironment in sarcopenia [14,15]. Unlike mononuclear cells, skeletal muscle fibers are multinucleated syncytia. Consequently, cell death in this context is often segmental [16]. It typically affects individual myonuclei or localized sarcomeric regions and does not immediately eliminate the entire fiber [16]. This distinction is essential for understanding how damaged muscle persists in an atrophic state rather than undergoing wholesale destruction. This article aims to systematically analyze the molecular mechanisms of various types of cell death in skeletal muscle diseases. We further summarize advances in cell death-related biomarkers and targeted therapies, aiming to establish a theoretical framework for diagnosis and treatment, and propose future research priorities.

## 2. Characteristics of Skeletal Muscle Disorders

### 2.1. Definition and Classification of Skeletal Muscle Disorders

Muscular disorders are a group of diseases caused by various factors, characterized by abnormal muscle structure and function, including muscle fiber damage, atrophy, or metabolic disorders. They usually present symptoms such as decreased motor ability, increased fatigue, and metabolic imbalance. Muscular disorders are mainly classified into two categories based on their causes and pathological features: hereditary and acquired. According to the causes and pathological features, muscular disorders include the following types [17]. Genetic disorders include DMD, GSD (such as Pompe disease), mitochondrial myopathies, etc. Acquired disorders cover idiopathic inflammatory myopathies, sarcopenia, rhabdomyolysis, endocrine myopathies (thyroid myopathy), toxic/drug-induced myopathies, and MG, etc. In addition to this etiological classification, skeletal muscle disorders can be broadly divided into primary myopathies and neurogenic disorders. In primary myopathies, the pathological process originates within the muscle fiber itself. In neurogenic disorders, muscle atrophy and dysfunction arise secondary to impaired innervation or neuromuscular transmission. MG is a classic example of the latter [18].

DMD is the most common form of muscular dystrophy [19], primarily caused by mutations in the dystrophin gene, and eventually leads to muscle degeneration and related complications [20,21,22,23]. Without dystrophin, muscle fibers are prone to damage during contraction, ultimately resulting in muscle atrophy and weakness.

GSD is a group of genetic metabolic disorders caused by mutations in genes related to glycogen metabolism. Deficiency of the related enzyme activities leads to disorders in glycogen synthesis and decomposition, resulting in abnormal glycogen deposition in multiple organs throughout the body. Based on the type of enzyme defect and the affected tissues, it can be classified into 16 subtypes, with skeletal muscle involvement being the most common, known as glycogen storage myopathy, which is more commonly seen in types II and V. Among them, GSD type II is also known as Pompe disease, acid alpha-glucosidase deficiency, acid maltase deficiency (AMD), and is an autosomal recessive genetic disorder. Due to the mutation of the acid alpha-glucosidase (*GAA*) gene, the degradation of glycogen in lysosomes is impaired, causing abnormal accumulation of glycogen in skeletal muscles, cardiac muscles and the central nervous system [24]. According to the age of onset, severity of the disease, progression speed and affected tissues and organs, Pompe disease can be classified into infantile-onset disease (infantile-onset Pompe disease, IOPD) and late-onset disease (late-onset Pompe disease, LOPD) [25].

Mitochondrial diseases are a group of genetic disorders that result from mutations in mitochondrial DNA (mtDNA) or nuclear DNA (nDNA), causing oxidative phosphorylation (OXPHOS) dysfunction, ultimately leading to abnormal mitochondrial structure and function, and subsequently affecting multiple systems. When the skeletal muscles are primarily affected, it is called mitochondrial myopathy (MM). If the central nervous system is also affected simultaneously, it is called mitochondrial encephalomyopathy [26]. Among them, mitochondrial encephalopathy, lactic acidosis and stroke-like episodes (MELAS) is the most common clinical type. Muscle biopsy is one of the important auxiliary examination methods for mitochondrial myopathy. The results of muscle and nerve biopsy show characteristic fragmented red fibers (ragged red fibers, RRFs) and a large accumulation of degenerated mitochondria [27].

Idiopathic inflammatory myopathy (IIM) is a heterogeneous autoimmune muscle disorder of unknown etiology. It includes polymyositis (PM), dermatomyositis and inclusion body myositis (IBM), which are characterized by the infiltration of inflammatory cells into muscle tissue, leading to muscle weakness [28]. Hallmarked by inflammatory cell infiltration into muscle tissue, all subtypes cause progressive weakness. Unlike other types of IIM, IBM represents an age-associated disorder with twofold higher male predominance and resistance to immunosuppressive therapies [29]. Beyond inflammation, IBM pathognomonically features autophagic vacuole accumulation and protein aggregates (e.g., amyloid-β peptide, phosphorylated tau, p62, TDP-43) [30].

Sarcopenia is a degenerative and systemic muscle disorder. Its development is related to age, nutrition, physical exercise, and other diseases, as well as mitochondrial dysfunction, oxidative stress, inflammation, and many other pathways [31]. Histologically, it exhibits dystrophic changes (predominantly in type II fibers), fatty infiltration, progressive fibrosis, and diminished satellite cell populations [32].

RML is a well-known clinical syndrome of muscle injury caused by skeletal muscle cell damage and the release of intracellular components, such as myoglobin and creatine kinase (CK), into the systemic circulation [8,33,34]. Characteristic features include myalgia, weakness, and dark tea-colored urine, with clinical severity ranging from asymptomatic enzyme elevation to acute kidney injury [8].

MG is an acquired autoimmune disease of the nervous system, characterized by the presence of autoantibodies against certain proteins of the postsynaptic membrane at the neuromuscular junction, which leads to fluctuating muscle weakness [35]. Currently, the pathogenic antibodies involved in the pathogenesis of MG include acetylcholine receptor antibody (AChR-Ab), muscle-specific receptor tyrosine kinase antibody (MuSK-Ab), and low-density lipoprotein receptor-related protein 4 antibody (LRP4-Ab), among others [36].

Despite distinct pathophysiological mechanisms and clinical presentations, most skeletal muscle disorders share fundamental involvement of dysregulated cell death and aberrant tissue remodeling processes.

### 2.2. Pathogenic Features of Skeletal Muscle Disorders

The occurrence and development of skeletal muscle diseases is a complex process driven by intrinsic genetic defects and external environmental factors, and forms a vicious cycle through chronic inflammation and pathological microenvironment. First of all, genetic and molecular defects constitute the inherent conditions for the occurrence and development of diseases. For example, in DMD, mutations in the dystrophin gene lead to the loss of structural protein complexes of the muscle membrane, causing secondary metabolic disorders such as calcium influx and mitochondrial dysfunction, leading to muscle fiber fragility, necrosis, and fibrosis [2,37,38].

Secondly, external environmental factors can induce or exacerbate the pathological process. These include direct physical insults (such as mechanical trauma), as well as oxidative stress triggered by states such as aging and chronic diseases. Excessive production of reactive oxygen species (ROS) damages tissues, induces cell death, and further worsens the pathological process by oxidizing proteins, lipids, and DNA [39,40,41].

More importantly, the intrinsic defects and external injuries will add up to each other, so that the inflammation and abnormal internal environment of the muscle continue to worsen, forming an increasingly serious vicious circle. Following acute injury in healthy skeletal muscle, the process of degeneration and regeneration is coordinated by a local inflammatory response in which immune cells such as T cells, macrophages, and neutrophils infiltrate the muscle and release high levels of proinflammatory cytokines, clearing the damage [42]. In the context of diseases with intrinsic defects, such as DMD, chronic inflammation leads to excessive ROS levels in muscle, resulting in lipid peroxidation of muscle cell membranes and oxidative modification of proteins, which further leads to muscle atrophy, inhibits the regenerative capacity of satellite cells, and affects muscle regeneration [42,43]. This chronic inflammatory microenvironment persistently releases high levels of proinflammatory cytokines (e.g., TNF-α, IL-1β, IL-6) and chemokines, which not only directly attack muscle fibers but also actively induce or exacerbate various types of regulatory cell death through multiple mechanisms [44,45,46,47,48,49].

Beyond the biochemical crosstalk of cytokines, the extracellular matrix (ECM) serves as a critical biomechanical checkpoint in the progression toward cell death. For example, in DMD, pathological fibrosis leads to excessive deposition of ECM components such as collagen, resulting in increased tissue stiffness [50]. The sclerotic extracellular matrix produces an abnormal stretch on the sarcolemma during muscle contraction, continuously activating the mechano-sensitive ion channel Piezo1, leading to increased calcium influx [51,52]. Calcium overload in muscle cells induces the continuous opening of mitochondrial permeability transition pore (mPTP), which leads to mitochondrial dysfunction, the production of ROS and the release of cytochrome C [53,54], and finally activates the apoptosis signaling pathway, leading to muscle fiber damage. At the same time, ECM deposition promotes the nuclear translocation and activation of transcriptional coactivators YAP/TAZ [55]. The continuous activation of YAP/TAZ mainly drives the expression of pro-fibrosis and cell proliferation-related genes (such as *CTGF* and *CYR61*), which aggravates collagen deposition and fibroblast accumulation and further deteriorates the matrix microenvironment [55,56]. On this basis, a YAP/TAZ-driven fibrotic microenvironment may aggravate oxidative stress levels in muscle fibers, together with mitochondrial ROS burst caused by mPTP opening, and cooperate with other signaling pathways to promote muscle fiber injury and death.

As the pathological processes described above develop, a related question arises: is death confined to specific myonuclei in this syncytial structure, or must the entire muscle fiber be destroyed? In the syncytial structure of skeletal muscle fibers, apoptosis can be initiated from a localized region of the muscle fiber, often resulting in the loss of a single myonucleus and not immediately leading to the structural collapse of the entire muscle fiber [57]. In addition, under pathological conditions, such as DMD, the stability of the sarcolemma is destroyed due to the loss of dystrophin, triggering a series of pathological processes such as calcium overload, increased ROS, chronic inflammation and ECM deposition, which eventually leads to repeated damage of muscle fibers and persistent mynucleus loss [58]. When the loss of myonuclei exceeds a certain limit, leading to the imbalance of the nucleoplasm ratio, the repair ability of the remaining myonuclei is insufficient to maintain the normal function of the fiber, which may eventually lead to the cell death of the entire muscle fiber.

## 3. Roles and Mechanisms of Cell Death in Skeletal Muscle Disorders

### 3.1. Classification of Cell Death

Cell death is fundamentally categorized into accidental cell death (unregulated, passive) and regulated cell death (RCD) [59,60], the latter includes apoptosis, necroptosis, autophagic cell death, pyroptosis, ferroptosis, copper death, disulfide death, NETosis and other molecularly regulated pathways. Each RCD modality is characterized by distinct triggers, signaling cascades, and effector mechanisms. A brief overview of the core execution machinery for each pathway is provided in the respective cell death type-specific sections below, where their pathophysiological relevance to skeletal muscle is discussed in detail.

### 3.2. The Role of Apoptosis in Skeletal Muscle Diseases

Apoptosis is a non-lytic, caspase-dependent form of programmed cell death characterized by cell rounding, cytoplasmic condensation, and increased nuclear chromatin density [61,62,63]. During this process, the plasma membrane invaginates to form bud-like protrusions around chromatin fragments and organelles, which gradually separate into apoptotic bodies and are cleared by phagocytes [61,62,63]. This tightly regulated process plays an essential role in development and tissue homeostasis. In contrast, skeletal muscle fibers, as multinucleated syncytia, do not undergo whole-cell fragmentation. Apoptosis here typically manifests as selective myonuclear apoptosis—the deletion of individual nuclei within an otherwise intact fiber segment—rather than classical apoptotic body formation [16].

**Figure 1:**
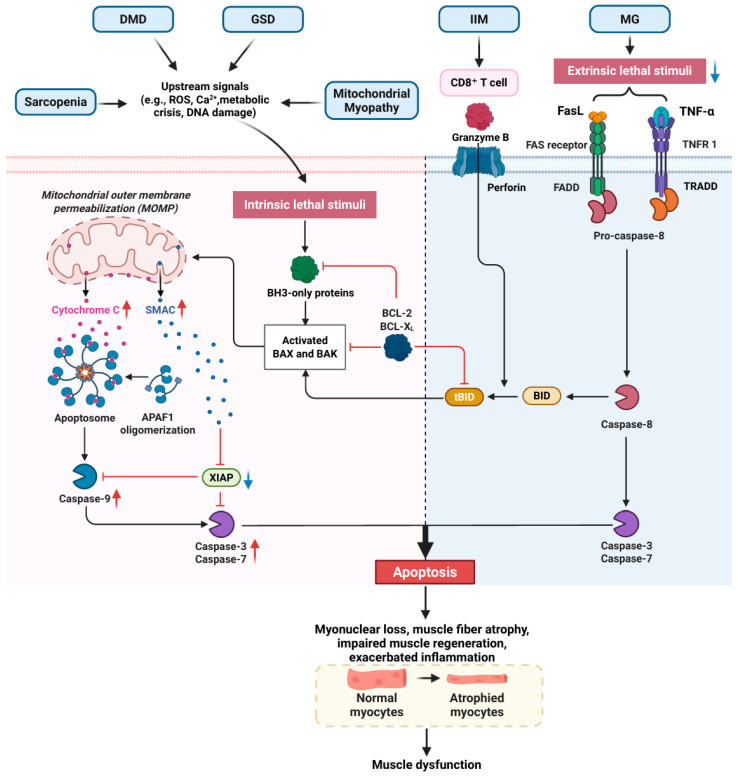
**Apoptosis in skeletal muscle diseases.** The intrinsic (mitochondrial) pathway is triggered by diverse intracellular stresses prevalent in skeletal muscle pathology, including ROS, Ca^2+^ overload (e.g., in DMD), metabolic crisis, and DNA damage. These insults disrupt the balance of Bcl-2 family proteins, promoting Bax/Bak oligomerization and Mitochondrial Outer Membrane Permeabilization (MOMP). Cytochrome c release facilitates apoptosome formation and Caspase-9 activation, while second mitochondria-derived activator of Caspases (SMAC) release antagonizes XIAP. This pathway contributes to the pathogenesis of sarcopenia, Duchenne muscular dystrophy (DMD), glycogen storage diseases (GSD), and mitochondrial myopathies. The extrinsic (death receptor) pathway is initiated by ligands (e.g., FasL from CD8^+^ T cells, TNF-α) binding to cognate receptors (Fas, TNFR1). This recruits adaptor proteins (FADD, TRADD) to form signaling complexes, leading to activation of initiator Caspase-8. In myasthenia gravis (MG), this pathway, especially Fas/FasL, often has functional defects or abnormal expression in peripheral autoreactive T cells, leading to the block of immune cell apoptosis and clearance, which is an important link in the initiation and maintenance of MG autoimmunity. In idiopathic inflammatory myopathy (IIM), myofiber apoptosis is mainly directly triggered by CD8^+^ T cells through the granzyme B-perforin pathway. Both pathways converge on the activation of executioner Caspases-3/7, culminating in apoptotic cell death. In skeletal muscle, this results in myonuclear loss, muscle fiber atrophy, impaired muscle regeneration, exacerbated inflammation, and functional decline, which are common hallmarks across multiple skeletal muscle diseases. Black arrows indicate promotion or activation. Blunt-end red arrows indicate inhibition. Red arrows indicate upregulation. Blue arrows indicate downregulation. Figure created with BioRender.com (https://biorender.com/q5c5uri).

Apoptosis proceeds via two principal pathways: the intrinsic (mitochondrial) pathway, triggered by intracellular stresses and regulated by Bcl-2 family proteins leading to cytochrome c release and caspase-9 activation [64], and the extrinsic (death receptor) pathway, initiated by ligand-bound death receptors and propagated through caspase-8 [65,66]. Both converge on executioner caspases-3/7 to dismantle the cell. However, its dysregulation has become a central aspect of the pathological processes of various skeletal muscle diseases, leading to the loss of muscle fibers and functional impairment. These diseases include sarcopenia, IIM (such as polymyositis and IBM), DMD and MG. Despite differences in the pathogenesis of various skeletal muscle disorders, apoptosis serves as a common pathway that significantly exacerbates muscle degeneration (Figure 1).

Sarcopenia is characterized by the loss of muscle mass and strength associated with aging [6]. Recent studies have shown that the activation of apoptotic signaling pathways is a key aspect of the pathogenesis of age-related sarcopenia [61,67,68]. Firstly, ROS accumulation and inflammatory factors (such as TNF-α and IL-1β) act as apoptotic stimuli and directly trigger myonucleus apoptosis by activating mitochondrial pathway and death receptor pathways, leading to muscle fiber atrophy, satellite cell depletion and accelerated protein degradation, thereby exacerbating sarcopenia degeneration [67,68,69,70,71]. In addition, the central concept, the “nuclear apoptosis” hypothesis, proposes that multinucleated skeletal muscle fibers selectively lose a portion of their myonuclei through apoptosis, leading to muscle fiber atrophy [61]. This process is associated with an imbalance in the expression of apoptosis-related proteins. For instance, in the skeletal muscle of aged rats, the expression of the pro-apoptotic factor Bax is increased, while the anti-apoptotic factor Bcl-2 is decreased, resulting in an imbalance in the Bax/Bcl-2 ratio and activation of the mitochondrial apoptotic pathway [72].

The decline in AMPK activity may exacerbate the progression of sarcopenia. Reduced AMPK phosphorylation levels lead to increased apoptosis in differentiating myoblasts, whereas activation of AMPK by AICAR suppresses the expression of apoptotic markers (e.g., Caspase-3, Bax) and maintains the number of myonuclei [73]. Collectively, these studies suggest that apoptosis directly contributes to the development of sarcopenia by reducing the number of myonuclei and disrupting mitochondrial function. The FOXO3 transcription factor plays a dual role in sarcopenia. It not only promotes the expression of key genes in the ubiquitin–proteasome system (e.g., *Atrogin-1*, *MuRF-1*), thereby promoting protein degradation, but also upregulates the expression of pro-apoptotic genes (e.g., *Bim*), directly contributing to muscle fiber apoptosis [74].

In addition, the Pardo team reported the mechanism of anti-apoptotic miRNA (miR-434-3p)/eukaryotic translation initiation factor 5A1 (eIF5A1) axis regulating skeletal muscle aging-related apoptosis [68]. miR-434-3p expression is significantly decreased in skeletal muscle during aging, leading to up-regulation of its target gene eIF5A1 [68]. eIF5A1, as a pro-apoptotic protein, activates the mitochondrial (intrinsic) apoptotic pathway, which is characterized by inducing loss of mitochondrial membrane potential and promoting activation of the apoptotic execution protein caspase-3/8/9, thereby driving myocyte apoptosis [68]. Overexpression of miR-434-3p or direct eIF5A1 knockdown inhibited cell death triggered by apoptosis inducers (TPEN/staurosporine) in myotubes [68]. In the muscle tissues of aging mice, a negative correlation between the low expression of miR-434-3p and the high expression of eIF5A1 was also observed [68]. These findings suggest that aging-associated downregulation of miR-434-3p increases eIF5A1 expression, which in turn overactivates the mitochondrial apoptotic pathway, leading to muscle fiber loss and sarcopenia. Therefore, miR-434-3p/eIF5A1-regulated apoptosis may be involved in the initiation and progression of sarcopenia.

The process of apoptosis is commonly observed in diseases classified under IIM, such as PM and IBM. Muscle biopsies of IIM show significant inflammatory cell infiltration, primarily involving T cells (CD3, CD8) and macrophages (CD68) [75]. Specifically, in muscle biopsies from PM and IBM patients, MHC I-expressing muscle fibers are invaded by inflammatory cells, and this invasion is dominated by CD8^+^ cytotoxic T cells [76]. This condition is often referred to as partial invasion and is included in the classification criteria of PM [77]. The inflammatory cell-infiltrated fibers did not show typical signs of necrosis but were gradually disintegrated and displaced by inflammatory cells [76]. In IIM with partial invasion (mainly PM and IBM), CD8^+^ T cells mediate two independent mechanisms of muscle fiber damage. One is the disintegration of muscle fibers through direct infiltration (partial invasion), and the other is the induction of apoptosis in adjacent muscle fibers that have not been infiltrated by immune cells by releasing effector molecules such as granzyme B [78]. These two mechanisms are spatially coexistent but target distinct populations of myofibers, representing two independent pathways of fiber degeneration.

During the progression of DMD, skeletal muscles initially exhibit weakness and fibrosis-induced atrophy [79], followed by apoptosis of muscle cells [80,81]. The lack of dystrophin leads to reduced sarcolemmal integrity, resulting in calcium overload and mitochondrial dysfunction [82]. This activates the Bax/Bak-dependent mitochondrial apoptotic pathway, whose central role lies in the regulation of mitochondrial outer membrane permeability (MOMP) to release cytochrome C, which in turn activates the Caspase cascade. In a mouse model, Bax/Bak double-knockout inhibited the mitochondrial apoptotic pathway by preventing the increase in MOMP, thereby protecting skeletal muscle cells from apoptosis induced by stress-induced injury, such as ischemia–reperfusion, and alleviating secondary inflammatory responses [83]. Mitochondrial damage leads to excessive production of reactive ROS, inducing oxidative stress and further activating apoptosis, forming a self-amplifying vicious cycle of “mitochondrial dysfunction–ROS–apoptosis” [84,85,86,87]. This severely compromises mitochondrial integrity and function [88], accelerating muscle weakness and atrophy. The persistent chronic inflammatory environment in DMD also significantly exacerbates apoptosis [89]. Ultimately, excessive apoptosis results in muscle cell loss that exceeds the capacity of satellite cells to repair and to regenerate muscle fibers, creating an “apoptosis–regeneration imbalance” [90]. Notably, although the NF-κB pathway exerts anti-apoptotic effects in myofibroblasts, its persistent activation in muscle fibers promotes inflammation, further exacerbating the apoptosis–regeneration imbalance [91].

The specific mechanism of apoptosis in GSD is still unclear, but existing studies have shown that apoptosis may play an important role in the pathogenesis of LOPD. LOPD patients usually develop the disease after the age of one, and their muscle pathological features include varying degrees of vacuolar degeneration of muscle fibers and glycogen deposition, with eosinophilic granule cells appearing in some muscle fibers and positive PAS staining, and acid phosphatase (ACP) stain indicating enhanced acid phosphatase activity in vacuolar degenerated muscle fibers [92]. Studies have found that as the age of Pompe disease model mice (Gaa^−/−^) increases, the pro-apoptotic p53 signaling pathway in their spinal cords significantly upregulates, and TUNEL staining confirmed the apoptosis of motor neurons [93]. In addition, through immunohistochemical and immunofluorescence analysis of 10 LOPD patients’ skeletal muscle biopsy samples, it was found that the expression of apoptosis-related proteins (such as Caspase-8, Cathepsin B) has specific distribution characteristics [94]. Caspase-8, as an important trigger factor of the extrinsic apoptotic pathway, is highly expressed in the early lesion areas with mild muscle fiber damage, such as the core area that has not vacuolated or only has punctate/vesicular changes. This suggests that the Caspase-8-mediated extrinsic apoptotic pathway is activated early in the disease and may drive the progression of muscle fibers from early lesions to late vacuolated necrosis. Cathepsin B is one of the tissue proteases with high content in lysosomes. After lysosomal membrane permeability is increased, Cathepsin B is released into the cytoplasm and triggers apoptosis by cleaving downstream signaling molecules, but the specific target protein is still unclear [26,95].

The core role of apoptosis in mitochondrial myopathy is executed through the mitochondrial apoptotic pathway (the intrinsic pathway), which is triggered by mitochondrial dysfunction and involves abnormal opening of the mitochondrial mPTP, the release of pro-apoptotic factors, and the activation of the Caspase cascade. The molecular mechanism begins with OXPHOS defects caused by mitochondrial DNA or nuclear gene mutations, and the dysfunction of the electron transport chain leads to excessive accumulation of mitochondrial reactive oxygen species (mtROS) [96]. When mtROS accumulates beyond a certain threshold, it will intensify oxidative stress and interact with the calcium ion (Ca^2+^) homeostasis imbalance, jointly inducing a decrease in mitochondrial membrane potential and causing the continuous opening of mPTP [97,98].

The mPTP is a complex structure spanning the Inner Mitochondrial Membrane (IMM) and Mitochondrial Outer Membrane (OMM), including voltage-dependent anion channel (VDAC), adenine nucleotide translocator (ANT), ATP synthetase, and cyclophilin D [99]. The prolonged opening of the mPTP can lead to a secondary burst of ROS, a process known as ROS-induced ROS release (RIRR) [97]. Eventually, this results in the collapse of mitochondrial membrane potential and an increase in the permeability of the IMM. Additionally, the high-level ROS upregulates pro-apoptotic proteins (such as Bax) and inhibits anti-apoptotic proteins (such as Bcl-2), leading to an increase in MOMP. When MOMP is increased and mPTP remains open, apoptotic factors such as cytochrome C, SMAC, and mtDNA will be released into the cytoplasm. Cytochrome C interacts with APAF1 to form an apoptotic body, activating Caspase 9, which in turn activates Caspase 3 and Caspase 7, ultimately driving the apoptotic process. Moreover, SMAC enhances the Caspase cascade process by inhibiting X-linked inhibitor of apoptosis protein (XIAP) [100]. Multiple studies have confirmed that there is an obvious phenomenon of cell apoptosis in the skeletal muscles of patients with mitochondrial myopathy. The Monici team conducted muscle biopsies on 10 patients with mitochondrial myopathy using TUNEL staining, and found significant nuclear apoptosis in the muscle samples of mitochondrial myopathy patients (71 ± 17% of muscle fibers showed DNA fragmentation) [101]. The Aure team analyzed the muscle samples of 20 patients and found that apoptosis occurred only in the muscle fibers with abnormal proliferation of mtDNA, and the main feature of this mtDNA amplification was the appearance of RRF. The study also found that this proliferation simultaneously increased both normal and mutant mtDNA, but did not alter their proportions. Moreover, the degree of apoptosis was more severe in RRFs with a higher mutation load [102]. This indicates that the fundamental cause triggering apoptosis is not the proliferation of mitochondria itself, but rather the severe mitochondrial dysfunction resulting from the high mutation load.

Mutations in *OXA1L* can lead to severe mitochondrial myopathy. OXA1L (Oxidase Assembly 1-like protein) is located in the inner mitochondrial membrane and acts as a mitochondrial membrane protein insertion enzyme, playing a crucial role in the assembly of the respiratory chain complex (CI-V). The Jang team discovered that patients with *OXA1L* gene mutations have OXPHOS disorders. In studies using myotube cells differentiated from patient-derived induced pluripotent stem cells (hiPSC), *OXA1L* knockout immortalized skeletal muscle cells (IHSMC), and skeletal muscle-specific *OXA1L* conditional knockout mouse models, it was demonstrated that *OXA1L* deficiency causes abnormal assembly and function of mitochondrial respiratory chain complexes (CI, CIII, CIV, CV), leading to OXPHOS disorders [103]. The study found that OXPHOS defects induce excessive accumulation of mitochondrial ROS, activating the NF-κB signaling pathway. This pathway, on the one hand, inhibits the expression of myogenic differentiation genes (such as *MYBPC2*, *MEF2C*), hindering the formation of myotubes; on the other hand, it upregulates pro-apoptotic factors (such as *TNFRSF1B*), activating Caspase-dependent apoptosis. Using the ROS scavenger Mito-TEMPO, apoptosis can be significantly reduced and muscle strength can be improved, confirming that the ROS-NF-κB axis is the core mechanism of OXA1L deficiency-mediated pathogenesis. However, the downstream molecules of NF-κB regulating apoptosis have not been fully elucidated.

In the pathogenesis of MG, although abnormal apoptosis mainly occurs in immune cells rather than muscle fibers themselves, the immune dysregulation caused by apoptosis can eventually indirectly lead to muscle dysfunction. In the thymus, the decrease in the apoptosis of autoreactive T cells in MG patients is mainly related to Bcl-2 overexpression and abnormal Fas/FasL pathway (such as decreased Fas expression or gene mutation) [104,105]. The arrest of apoptosis leads to abnormal accumulation of autoreactive T cells in the thymus and promotes the production of AChR antibodies, which attack the neuromuscular junction (NMJ), resulting in muscle weakness. In the peripheral immune system, CD4^+^ T cells also have apoptotic disorders [106], which is related to the block of Fas-mediated apoptotic pathway. Glucocorticoid treatment could accelerate the clearance of pathological immune cells by promoting apoptosis of CD4^+^ T cells and up-regulating Fas expression [106]. Therefore, although apoptosis mainly occurs in immune cells rather than muscle cells, abnormal apoptosis of immune cells leads to immune dysfunction, driving autoantibody production and neuromuscular junction destruction, which will indirectly cause muscle dysfunction.

In conclusion, apoptosis plays a crucial role in the pathological processes of various skeletal muscle diseases. Although the causes and pathological manifestations of different diseases vary, the dysregulation of apoptosis collectively constitutes an important pathway for muscle fiber loss and functional impairment. In sarcopenia, apoptosis leads to myonuclear reduction and mitochondrial dysfunction through nuclear apoptosis, and is closely related to abnormal signaling networks such as FOXO, AMPK, and miRNA. In IIM, T cell-mediated immune responses synergistically induce apoptosis and ER stress, a process that further exacerbates muscle fiber damage. In DMD, dystrophin deficiency triggers calcium overload, oxidative stress, and mitochondrial dysfunction, continuously activates the mitochondrial apoptosis pathway, and eventually leads to the progressive loss of muscle fiber and function. In GSD (such as LOPD) and mitochondrial myopathies, apoptosis is significantly activated through lysosomal and mitochondrial pathways, and the metabolic defects and oxidative stress caused by mutant genes further drive this process. In MG, abnormal apoptosis is mainly manifested as the obstruction of the clearance of immune cells (such as thymocytes and peripheral T cells), resulting in the accumulation of autoreactive lymphocytes and the continuous progression of autoimmune reactions. Given that apoptosis is a common pathological process in various skeletal muscle diseases, interventions targeting specific apoptotic pathways or key molecules, such as by regulating the activity of key Caspases [107] have shown potential as therapeutic strategies to reduce muscle fiber loss and delay disease progression. It can improve the clinical prognosis of a variety of skeletal muscle diseases.

### 3.3. The Role of Necroptosis in Skeletal Muscle Diseases

Necroptosis is a programmed and inflammatory form of cell death, which is mediated by Receptor-Interacting Protein Kinase 1 (RIPK1) and Receptor-Interacting Protein Kinase 3 (RIPK3), as well as Mixed Lineage Kinase Domain-Like protein (MLKL) [108]. Its initiation depends on the inhibition of the apoptosis pathway (such as inactivation of Caspase-8) [109]. When death ligands bind to the corresponding receptors, such as TNF-α binding to TNFR1, RIPK1 interacts with RIPK3 via the RHIM domain to form an amyloid-like filamentous complex (necrosome). This complex phosphorylates MLKL, inducing conformational changes that lead to MLKL oligomerization and insertion into the plasma membrane to form pores. This causes the release of DAMPs (such as HMGB1 and ATP), activating the TLR4/NF-κB pathway or the NLRP3 inflammasome, triggering an immune response. This forms a pro-inflammatory positive feedback loop, ultimately leading to cell death.

**Figure 2:**
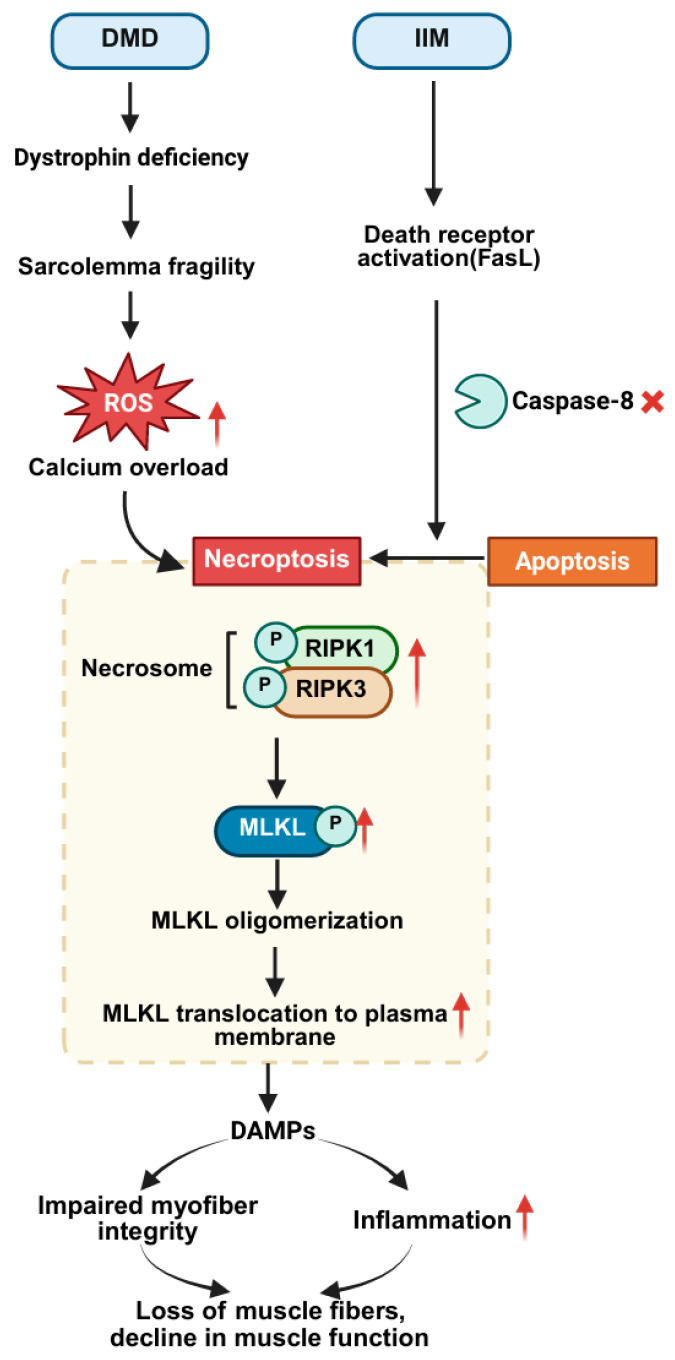
**Necroptosis in skeletal muscle diseases.** In DMD, dystrophin deficiency leads to sarcolemma fragility, resulting in pathological calcium influx and subsequent mitochondrial dysfunction. This triggers reactive oxygen species (ROS) overproduction and calcium overload, which converge to activate the necroptosis pathway. Concurrently, inflammatory cytokines released in the dystrophic microenvironment further potentiate death receptor signaling. In IIM, activated CD8^+^ T cells express death receptor ligands (e.g., FasL). Activation of death receptors (e.g., FasL binding to Fas) on muscle fibers initiates the extrinsic apoptosis pathway. However, under conditions where Caspase-8 activity is suppressed, the signal diverts from apoptosis to necroptosis. Signals from both disease contexts lead to the phosphorylation and activation of RIPK1 and RIPK3, which form the necrosome complex. This complex phosphorylates MLKL, inducing its oligomerization and translocation to the plasma membrane. MLKL pores disrupt membrane integrity, causing the release of damage-associated molecular patterns (DAMPs). DAMP release initiates a potent pro-inflammatory response, recruiting immune cells and forming a pathogenic positive feedback loop that exacerbates tissue damage. The execution of necroptosis directly impairs myofiber integrity, culminating in the loss of muscle fibers and a progressive decline in muscle function. Black arrows indicate promotion or activation. Red arrows indicate upregulation. A cross mark (“X”) indicates inhibition. Figure created with BioRender.com (https://biorender.com/q5c5uri).

Necroptosis plays a critical role in the pathological progression of DMD (Figure 2). The loss of dystrophin leads to membrane fragility, which predisposes muscle fibers to calcium overload and mitochondrial ROS bursts. These events activate the RIPK1/RIPK3/MLKL pathway, directly driving necroptosis in muscle fibers, increasing cell death, and exacerbating muscle dysfunction [110]. There is evidence that the expression of RIPK1, RIPK3, and MLKL is significantly elevated within muscle tissue in DMD models such as mdx mice, a finding that provides key evidence for necrotizing apoptotic pathway activation [111]. Functionally, RIPK3 gene knockout effectively reduces muscle fiber necroptosis, inflammatory infiltration, and fibrosis while improving muscle function in mdx mice [111]. Pharmacological inhibition of this pathway (e.g., using RIPK1 inhibitor Necrostatin-1 or MLKL inhibitor necrosulfonamide) also partially alleviates cell death and pathology [112], validating the potential of targeting necroptosis as a therapeutic strategy for DMD. However, single-pathway inhibition is insufficient to completely halt disease progression, suggesting a need for combined intervention targeting both necroptosis and other cell death or damage pathways [111,112].

Necroptosis is also critical in IIM types such as PM (Figure 2). In PM patients, muscle fiber death is characterized by FAS ligand-dependent necroptosis. Upon FAS activation, if Caspase-8 activity is inhibited (e.g., in a virus-infected or ROS-rich inflammatory microenvironment), the signaling shifts to RIPK1-dependent necroptosis [113]. This mode of cell death results in the release of DAMPs such as HMGB1, creating a positive feedback loop that further recruits and activates immune cells (e.g., CD8^+^ T cells), exacerbating muscle inflammation and damage [113]. Treatment with necroptosis inhibitors or anti-HMGB1 antibodies can alleviate muscle weakness, cell death, and inflammation caused by myositis [113]. This indicates that muscle cells undergoing necroptosis in PM actively participate in shaping the inflammatory microenvironment.

It is critical to understand the complexity of skeletal muscle cell death, in which necroptosis often coexists with other death mechanisms and is regulated by common upstream factors. Mitochondrial dysfunction serves as a central convergence point. ROS burst and energy crisis caused by oxidative stress and mitochondrial damage (common in DMD, inflammation, aging, etc.) are important triggers for the activation of RIPK1/RIPK3/MLKL-mediated necroptosis pathway [110]. Notably, the same conditions described above can also trigger other types of cell death, such as apoptosis. Therefore, in diseases like DMD, elevated necroptosis markers (e.g., RIPK3) are often accompanied by apoptosis (e.g., TUNEL-positive nuclei) [57,78,111,114,115], which reflects the crosstalk between different death pathways and the complex interactive dialogue among the internal death signaling pathways of the cells under stress conditions.

In conclusion, the mechanisms of necroptosis in skeletal muscle diseases exhibit multidimensional pathological features, and their dysregulation plays a critical role in muscle degenerative conditions. Given the central role of necroptosis in driving muscle degeneration and inflammation, targeting its key nodes (RIPK1, RIPK3, MLKL) shows therapeutic potential. In DMD [111,112]. and PM [113] models, blocking necroptosis pathway by RIPK3 gene knockout or specific inhibitors (RIPK1 inhibitor or MLKL inhibitor) has been shown to effectively reduce muscle pathological damage and improve function, proving the feasibility and effectiveness of targeting necroptosis as a potential treatment.

### 3.4. The Role and Mechanism of Autophagy-Related Cell Death in Skeletal Muscle Disorders

**Figure 3:**
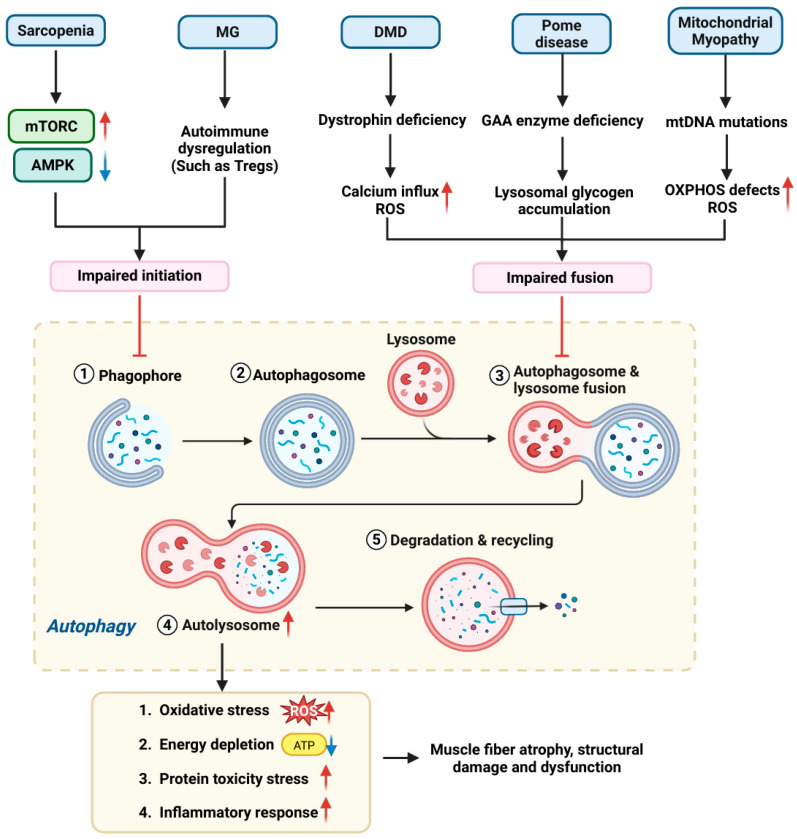
**Autophagy in skeletal muscle diseases.** Under normal conditions, cellular stress signals inhibit mTORC1 and activate AMPK, which induces autophagy initiation. The process involves phagophore formation, autophagosome maturation, cargo encapsulation, fusion with lysosomes to form autolysosomes, and subsequent degradation and recycling of cellular components (e.g., damaged mitochondria, protein aggregates). In sarcopenia, age-related increases in mTORC1 activity and decreases in AMPK activity primarily suppress autophagic initiation. In MG, impaired autophagy in immune cells (e.g., Tregs) contributes to autoimmune dysregulation. In contrast, DMD, Pompe disease (GSD II), and mitochondrial myopathies cause impairment of autophagosome–lysosome fusion and subsequent lysosomal degradation, often due to calcium/ROS overload, lysosomal dysfunction, or OXPHOS defects. Blockade of autophagic flux at any stage leads to the toxic accumulation of damaged organelles (e.g., swollen mitochondria) and protein aggregates. This triggers oxidative stress, energy depletion, proteotoxicity, and inflammation, which collectively drive myofiber atrophy, structural damage, and loss of contractile function. Black arrows indicate promotion or activation. Blunt-end red arrows indicate inhibition. Red arrows indicate upregulation. Blue arrows indicate downregulation. Figure created with BioRender.com (https://biorender.com/q5c5uri).

Autophagy is a process in which cells wrap cytoplasmic components (such as damaged organelles and proteins) by forming double-membrane autophagic vesicles and transport them to lysosomes for degradation to maintain cellular homeostasis and quality control [116,117]. This process requires autophagy-related genes (ATGs, such as *Beclin 1*, *ATG5*, *ATG7*) to coordinate autophagosome initiation and extension [118]. The key regulatory point for autophagy activation lies in the release of Beclin 1. Under normal conditions, Beclin 1 binds to the anti-apoptotic protein Bcl-2 and inhibits the initiation of autophagy. When subjected to pathological stimuli or specific drugs, this complex will dissociate, and free Beclin 1 can drive autophagic flow, thereby activating autophagy [119,120]. Either overactivation of autophagy or insufficient autophagy may disrupt cell homeostasis and trigger cell death. Recent studies have shown that autophagy-related cell death is closely associated with various skeletal muscle diseases such as DMD, sarcopenia, GSD and MG, and its mechanism of action shows disease type dependence (Figure 3).

The skeletal muscle autophagic system preserves contractile unit integrity through clearance of misfolded proteins (e.g., α-actinin aggregates) and damaged organelles (e.g., swollen mitochondria) [121]. Central to the induction of autophagy in most cell types is the downregulation of mTORC1 activity. When mTORC1 activity is inhibited under nutrient deprivation or stress, it is unable to further inhibit the ULK1-Atg13-FIP200 complex, which leads to the activation of ULK1 (unc-51-like autophagy activating kinase 1) complex to initiate autophagy [122]. In skeletal muscle, the activation of autophagy and the ubiquitin–proteasome system is not only regulated by mTORC1, but also depends on the FOXO3 transcription factor. FOXO3 drives muscle protein degradation by directly transactivating the expression of E3 ubiquitin ligases (e.g., atrogin1/MAFbx, MuRF1) and autophagy-related genes (e.g., *LC3*/*Bnip3*) [123,124].

Moderate autophagy helps to effectively remove and recycle damaged organelles and protein aggregates, thereby maintaining the homeostasis and function of muscle cells. However, impaired autophagy can lead to excessive degradation of myofibrils. For instance, in atrophic and functionally impaired skeletal muscles, the levels of autophagy-related markers increase [125]. Nevertheless, the muscle-specific deletion of autophagy regulators (such as ATG7, ATG5) can induce skeletal muscle atrophy [126]. Paolini et al. investigated the phenomenon that weakened autophagy leads to a reduction in the size of skeletal muscle fibers, as well as a decline in muscle regeneration capacity after starvation stress and acute injury [127]. The Atg16L1 hypomorph model (with partial autophagic suppression) revealed reduced myofiber cross-sectional area versus wild-type, indicating an important role of autophagy in muscle growth regulation. Under fasting conditions, mutant mice exhibited accelerated muscle wasting within 12 h, correlating with diminished autophagic induction, heightened oxidative stress, and elevated protein ubiquitination.

In DMD, the impairment of autophagic flux is manifested as a disorder in the fusion of autophagosomes and lysosomes, resulting in abnormal accumulation of damaged mitochondria and protein aggregates, which forms a pro-inflammatory microenvironment and exacerbates the disintegration of muscle fibers [128]. This autophagic dysfunction synergizes with dystrophin deficiency-induced membrane fragility, establishing a self-perpetuating degenerative cycle.

Skeletal muscle autophagy is regulated by multiple signaling pathways, such as AMPK and mTOR. In aged skeletal muscle fibers, an abnormal increase in mTORC1 activity is often observed, which inhibits the protective autophagy process [129]. However, activation of AMPK inhibits mTORC1 and induces autophagy [130,131]. Studies have shown that in the aged skeletal muscle-specific AMPK gene knockout mice model, the loss of AMPK function exacerbates autophagy dysfunction and aging-related myopathy, characterized by decreased muscle strength, mitochondrial abnormalities, and accumulation of autophagic cargoes [130]. These results corroborate the critical protective role of AMPK against muscle aging. Therefore, impaired autophagy and mitochondrial dysfunction can form a vicious cycle in aging, synergistically promoting the death of muscle cells and the degeneration of muscle tissue.

In sarcopenia, there is a close connection between autophagy impairment, oxidative damage, and subsequent cell death. The association between autophagy and oxidative stress may be in a bidirectional regulatory state, as oxidative stress promotes autophagy, while increased autophagy reduces oxidative damage and enhances antioxidant capacity, thereby reducing oxidative stimulation [132]. However, mitochondrial dysfunction (characterized by decreased respiration and increased ROS production) can act as an upstream trigger, disrupting the autophagy homeostasis in skeletal muscle cells [133]. This kind of damage may manifest as a blockage in the autophagy process, thereby promoting oxidative stress to increase the risk of cells undergoing autophagy-related cell death.

In Pompe disease (GSD type II), mutations in the *GAA* gene lead to a lack of, or significant reduction in, GAA activity in lysosomes, preventing glycogen from being degraded and causing it to accumulate in the lysosomes of cells in tissues such as skeletal muscle, cardiac muscle, and smooth muscle. The traditional theory emphasizes that the accumulation of glycogen in lysosomes leads to lysosomal swelling and a decrease in membrane stability. Additionally, the mechanical stress generated by muscle contraction further exacerbates the loss of lysosomal membrane integrity, resulting in the leakage of contents (such as glycogen and hydrolases) into the cytoplasm, triggering muscle fiber damage [134]. With the establishment of Pompe disease animal models in recent years, the application of enzyme replacement therapy (exogenous supplementation of GAA to remove glycogen from lysosomes) [135], and the development of electron microscopy technology, the role of autophagy in the pathogenesis of Pompe disease has gradually gained attention.

STBD1 is a key receptor protein that mediates glycogen autophagy. Recent studies have elucidated its molecular mechanism. STBD1 recognizes glycogen through the CBM20 domain and recruits the autophagy initiation factor RB1CC1 (ULK complex) and ATG8 protein (especially GABARAPL1) via the LIR motif to initiate autophagosome formation [136]. However, in Pompe disease, the damage of the lysosomal structure leads to a decrease in the lysosomal membrane stability of the lysosomal membrane and the leakage of enzymes within the lysosome, which hinders the normal fusion of autophagosomes with lysosomes, leading to the accumulation of cargoes (including undegraded glycogen) in autophagosomes and the formation of abnormal autophagic vesicles. Notably, type II muscle fibers (fast contracting muscle fibers), which rely on glycogenolysis for energy, are more susceptible to impaired autophagy in Pompe disease caused by *GAA* deficiency. When faced with energy stress signals triggered by glycogen energy supply impairment, myocytes may attempt to initiate autophagy as a compensation. Consistent with this, studies have shown that the expression of STBD1 is significantly increased in the skeletal muscle of Pompe disease model mice (GAA-KO) [137], which may be an adaptive response of the cells to accelerate the clearance of abnormally accumulated glycogen.

In mammalian cells, autophagy initiation depends on the activation of the most upstream regulatory factor ULK1 [138]. Under stress conditions such as starvation, AMPK can promote the initiation of autophagy by phosphorylating ULK1 (at Ser317 and Ser777 sites) [122]. At the same time, AMPK can inhibit the mTORC1 activity on the lysosomal surface through the TSC2/Rheb pathway, promoting the dissociation and inactivation of mTORC1 from the lysosome. After mTORC1 inactivation, the transcription factors TFEB/TFE3 undergo dephosphorylation and translocate into the nucleus, upregulating the expression of autophagy-related genes. However, in the pathological background where the lysosomal degradation function has failed, the activation of this upstream pathway will generate more autophagosomes that cannot be eliminated, which further aggravates the accumulation of autophagic vesicles and ultimately destroys the muscle fiber structure. Studies have found that in Pompe disease model mice, the core region of type II muscle fibers has large-scale autophagic accumulation (accounting for 40% of the muscle fiber volume), characterized by abnormal aggregation of LC3-positive autophagosomes and Lamp1-positive lysosomes [139]. This autophagic abnormality results from the disorder of autophagosome–lysosome fusion, accompanied by the upregulation of autophagy-related genes (such as *Beclin1*, *LC3*, etc.).

Importantly, autophagic accumulation directly damages the myofibrillar structure, disrupts the arrangement of fast myosin, and affects muscle contraction function. In the muscles of patients with Pompe disease, autophagy damage is also observed, with large vacuoles present in most muscle fibers, coexisting with a large amount of autophagic debris, damaging the muscle fiber structure and manifesting as the accumulation of LC3II, p62, and aggregates [140]. These features collectively confirm the severe damage to autophagic flux. In summary, Pompe disease is no longer simply regarded as a glycogen storage disease. The blockade of autophagic flow due to loss of lysosomal function and the resultant abnormal accumulation of autophagic vesicles conspire to cause trafficking dysfunction and structural destruction of myofibrils.

In mitochondrial myopathies, autophagy is particularly important because mitochondria are the energy factories of cells, and their dysfunction directly leads to insufficient energy supply and cell damage in muscle tissues. A study confirmed the dysfunction of mitochondrial autophagy (mitophagy) in mitochondrial myopathies [141]. This study showed that autophagy acts as a protective response to clear abnormal mitochondria in the early stages of the disease. At this time, the nucleus of muscle fibers moves toward the center (nuclear centralization), and mitophagy is enhanced in these regions, accompanied by the upregulation of lysosome biosynthesis markers. However, as the disease progresses, the mitochondria in RRFs swell and accumulate, the autophagy flow is stalled (mitolysosome decreases by 40–50%), accompanied by fragmentation of lysosomes, disordered spatial distribution, and accumulation of p62/SQSTM1. Mitolysosome is a degradation structure formed after the fusion of autophagosome and lysosome, which contains mitochondria that are being degraded, and is the key sign reflecting the completion of mitophagy flow. The significant reduction of this structure directly indicates that the autophagy process is blocked at the final degradation step. Unremoved damaged mitochondria produce excessive ROS, exacerbating mtDNA mutations and protein oxidation, thereby enhancing MOMP and continuous opening of mPTP, releasing cytochrome C and other pro-apoptotic factors, activating the Caspase cascade reaction, and ultimately leading to muscle cell apoptosis and atrophy.

In MG, a disorder characterized by autoantibody-mediated disruption of the NMJ, aberrant autophagy also contributes to skeletal muscle dysfunction. Studies have shown that autophagy activity is reduced in immune cells from MG patients, which may impair the clearance of autoreactive lymphocytes and thereby exacerbate autoimmune attack on the NMJ [142,143,144,145,146]. Furthermore, defective mitophagy at the NMJ leads to the accumulation of damaged mitochondria, resulting in insufficient ATP production and increased reactive oxygen species (ROS) [147]. These changes compromise acetylcholine synthesis, vesicle cycling, and postsynaptic membrane depolarization, ultimately disrupting nerve-muscle signal transmission and promoting muscle weakness. Thus, while the primary defect in MG is immunological and neural, autophagy deficiency in both immune cells and the NMJ microenvironment aggravates secondary skeletal muscle pathology.

Autophagy-related cell death is a complex process that significantly affects muscle health. Its effect is disease type and stage-specific, and is precisely regulated by multiple pathways such as mTOR, AMPK, and FOXO3. Specific knockout of AMPK in skeletal muscle inhibits autophagic flow and promotes muscle function decline and age-related muscle loss, whereas use of the mTORC1 inhibitor (Rapamycin) enhances autophagy in skeletal muscle of aged mice, improves mitochondrial respiration, and reduces age-related muscle loss [130,148]. It is worth noting that the disruption of the autophagic flux in skeletal muscle directly damages the structural integrity and physiological function of muscle fibers. For instance, knocking out Atg5 specifically in skeletal muscle completely blocks the autophagic flux (manifested as the inhibition of LC3 lipidation and the accumulation of p62 protein), resulting in mitochondrial abnormalities, muscle fiber atrophy, and decreased contractility [126]. Further studies are needed to elucidate the precise role of autophagy in different muscle pathologies and to develop targeted regulatory strategies.

### 3.5. The Role and Mechanism of Pyroptosis in Skeletal Muscle Diseases

**Figure 4:**
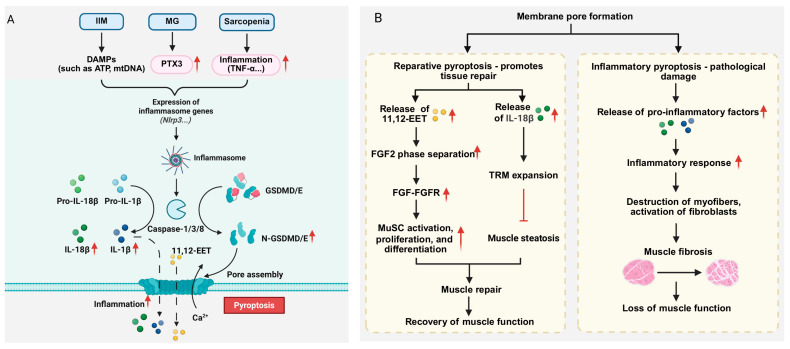
**Pyroptosis in skeletal muscle diseases.** (**A**) Disease-Specific Triggers and execution pathways. Schematic representation of pyroptosis induction in IIM, MG, and sarcopenia. In IIM, muscle fiber damage releases damage-associated molecular patterns (DAMPs, e.g., ATP, mtDNA), which are recognized by infiltrating myeloid cells (e.g., macrophages). This recognition leads to the expression and activation of the NLRP3 inflammasome in the macrophages themselves. In MG, the inflammatory mediator PTX3 is upregulated, which promotes inflammasome activation in myocytes via the STAT3 signaling axis. In sarcopenia, chronic inflammation associated with aging triggers pyroptosis signals within muscle cells. After stimulation by these triggers, the pathway leads to the activation of inflammatory caspases (Caspase-1/3/8), which cleave pro-IL-1β and pro-IL-18 into mature forms and cleave gasdermin proteins (GSDMD/E). The N-terminal fragments of GSDMD/E oligomerize to form pores in the plasma membrane, resulting in calcium ion (Ca^2+^) influx, the release of inflammatory cytokines, and execution of pyroptosis. (**B**) Pyroptosis exerts context-dependent effects. At the initial stage of muscle injury repair, highly activated macrophages selectively release the small-molecule lipid metabolite 11,12-EET through the GSDMD pore. This promotes liquid–liquid phase separation (LLPS) of fibroblast growth factor 2 (FGF2), enhancing FGF-FGFR signaling, which drives muscle stem cell (MuSC) activation, proliferation, and differentiation, ultimately leading to muscle repair and functional recovery. In addition, myeloid cells activate tissue-resident macrophages (TRM) via the GSDME/IL-18 axis to prevent muscle steatosis by inhibiting the differentiation of fibro-adipogenic progenitors (FAPs) into adipocytes, thereby promoting muscle regeneration. In the pathological environment, the formation of GSDMD pore leads to the release of pro-inflammatory factors (such as IL-1β, IL-18), triggering a strong inflammatory response, leading to muscle fiber destruction, fibroblast activation, muscle fibrosis, and ultimately loss of muscle function. Black arrows indicate promotion or activation. Blunt-end red arrows indicate inhibition. Red arrows indicate upregulation. Dashed arrows indicate release. Figure created with BioRender.com (https://biorender.com/q5c5uri).

Pyroptosis, a form of RCD activated by inflammasomes and executed by Gasdermin proteins (e.g., GSDMD, GSDME). It is triggered by inflammasome-mediated activation of inflammatory caspases, which cleave GSDMD to generate an N-terminal fragment that forms pores in the plasma membrane, facilitating the release of IL-1β, IL-18, and other cytosolic contents [149,150,151,152,153,154] (Figure 4A). Pyroptosis has shown pro-inflammatory and pro-repair roles in skeletal muscle diseases (Figure 4B). This duality is reflected in its ability to drive inflammation and exacerbate muscle pathology while promoting tissue repair under specific conditions.

The pro-inflammatory effect caused by pyroptosis is a central mechanism in driving the pathological progression of a variety of skeletal muscle diseases. DAMPs, such as ATP and mtDNA, released by pyroptotic cells can activate pattern recognition receptors (e.g., TLR9) in immune cells, stimulating the activation of myofibroblasts and thereby exacerbating muscle fibrosis and functional loss [155]. In inflammatory myopathies (e.g., polymyositis and IBM), pyroptosis is a key driver of the inflammatory cascade. The activation of the NLRP3 inflammasome in myeloid cells (such as macrophages and neutrophils) leads to the cleavage of GSDMD, resulting in the formation of cell membrane pores and the release of mature inflammatory factors IL-1β and IL-18 [156,157], significantly amplifying the local inflammatory response. Notably, in IBM, damaged muscle fibers release DAMPs such as mtDNA. These DAMPs are recognized by immune cells infiltrated in muscle tissue, such as macrophages, leading to the activation of NLRP3 inflammasome, which in turn triggers inflammatory response and oxidative stress, leading to muscle fiber damage and further deterioration of mitochondrial function. Concurrently, muscle tissues from IBM patients exhibit significantly elevated levels of phosphorylated Ubiquitin at Ser65 (p-S65-Ubiquitin), a specific marker of mitophagy that tags damaged mitochondria. This elevation suggests an altered mitophagy process, which may lead to the accumulation of damaged mitochondria due to enhanced autophagy initiation and degradation disorders [158]. The persistent release of DAMPs, such as mtDNA, from these damaged mitochondria activates the NLRP3 inflammasome, thereby establishing a self-perpetuating vicious cycle that exacerbates disease pathology. In addition, in IBM patients, *NLRP3* mRNA levels were positively correlated with p-S65-Ubiquitin levels in both men and women, suggesting that NLRP3 inflammasome activation is a pathological event shared by both sexes. However, further clinical association analysis showed that *NLRP3* mRNA levels were positively correlated with decreased muscle strength only in male IBM patients, suggesting that NLRP3 activation may play a more direct or critical role in driving muscle weakness in male patients than in female patients. Several studies have shown that patients with sarcopenia exhibit elevated levels of inflammatory markers and/or pro-inflammatory cytokines in their serum [159,160]. In the skeletal muscles of aged mice, the aging-related chronic low-grade inflammatory environment (characterized by elevated factors such as TNF-α) activates Caspase-8/3 in skeletal muscle cells, inducing GSDME cleavage and triggering pyroptosis of skeletal muscle cells. This process directly disrupts muscle fiber structure and causes muscle mass loss, while GSDME knockdown partially reverses muscle loss and reduces levels of inflammatory cytokines such as IL-6 and IL-1β [159]. The pro-inflammatory effects of pyroptosis primarily stem from the direct physical damage to muscle fibers caused by GSDM protein pore formation and the release of large amounts of pro-inflammatory mediators (e.g., IL-1β, IL-18), which contribute to the persistent deterioration of the muscle microenvironment [161].

Current studies have shown that pyroptosis, as a highly proinflammatory form of RCD, may participate in the pathological process of MG by amplifying inflammatory responses, causing tissue damage, and leading to muscle dysfunction. Studies have demonstrated that the expression of inflammatory NLRP3 is elevated in MG, and IL-1β can cause corresponding pathological changes [162]. Li et al. confirmed that the inflammatory mediator Pentraxin 3 (PTX3) is an important upstream driver of pyroptosis in MG [163]. This team found that the expression of PTX3 was significantly upregulated in serum of MG patients and in mouse models, and it enhanced the activity of the NLRP3 inflammasome by activating the STAT3 signaling pathway, promoting the activation of key mediators of pyroptosis (such as Caspase-1), and subsequently cleaving GSDMD, generating its activated form (N-GSDMD), which ultimately leads to the occurrence of cell pyroptosis and the release of a large amount of pro-inflammatory factors (such as IL-1β, IL-18), thereby exacerbating the inflammatory response and cell pyroptosis. Animal experiments confirmed that the recombinant protein of PTX3 can increase the level of AChR autoantibodies, promote the expression of chemokines (Ccl12/Ccl19/Ccl13) and pro-inflammatory factors (IL-1β, TNF-α, etc.), and enhance the pyroptosis process. In addition, the study also indicates that METTL3 enhances the stability of *PTX3* mRNA in myoblasts through m6A modification, further amplifying the inflammatory signal and enhancing the pro-pyroptotic effect of PTX3. These results reveal the core role of the PTX3/STAT3/NLRP3 axis in pyroptosis in MG, not only providing a new perspective for understanding the mechanism of inflammation amplification in MG, but also laying a theoretical foundation for the development of new therapeutic strategies and biomarkers for MG via targeting the pyroptosis process.

In stark contrast to its pro-inflammatory effects, pyroptosis plays an important beneficial role in muscle tissue repair. An increasing body of evidence suggests that specific pyroptosis signals mediated by proteins of the Gasdermin family are key to initiating and coordinating the effective repair of muscle tissue. Among them, the non-classical function of Gasdermin D (GSDMD) is one of the important pathways for mediating repair. The Chi group reported that in hyperactivated macrophages, the pores formed by GSDMD were able to selectively permeate and release small-molecule metabolites smaller than 3 kDa (such as 11, 12-epoxyeicosatrienoic acid, 11,12-EET) [161]. Myeloid cell-specific deletion of GSDMD was found to delay muscle regeneration, but did not affect local inflammation or pyroptosis. The 11,12-EET released by macrophages in a highly activated state enhances the liquid–liquid phase separation (LLPS) of fibroblast growth factor 2 (FGF2), thereby amplifying the signal transduction of the FGF-FGFR pathway. This mechanism ultimately promotes the activation, proliferation and differentiation of muscle stem cells (MuSCs) and accelerates the process of muscle regeneration. Notably, in aged mice and human muscles, the expression of EPHX2, an enzyme that degrades 11,12-EET, is upregulated, leading to a decrease in endogenous 11,12-EET levels and muscle regeneration disorders. In contrast, exogenous supplementation of 11,12-EET can improve this situation, as evidenced by the promotion of muscle stem cell activation, acceleration of myofiber repair after injury, and reduction of fibrosis [161]. On the other hand, recent studies have revealed the key role of Gasdermin E (GSDME) -dependent pyroptosis axis in inhibiting muscle steatosis and promoting muscle regeneration [164]. After skeletal muscle injury, the expression of GSDME is upregulated in monocyte-derived macrophages recruited to the injury site, and pyroptosis signal triggered by it promotes the release of cytokine IL-18. In turn, IL-18 drives the expansion of tissue-resident macrophages (TRMs) with a unique repair phenotype (Lyve1^+^ Cd163^+^ Txnip^+^) and maintains their function by activating transcription factors such as KLF4 and JUN. This “GSDME-IL-18-TRMs” axis can effectively inhibit the differentiation of fibro-adipogenic progenitors (FAPs) into adipocytes, thereby preventing muscle steatosis. Notably, this axis function is attenuated in aging models, whereas exogenous supplementation of IL-18 reverses steatosis and promotes muscle regeneration. These findings provide insights into intervention strategies for aging-associated muscle aplasia, one of the potential triggers of sarcopenia.

Given the dual roles of pyroptosis as both a destroyer and a repairer in skeletal muscle, targeting its distinct functional axes represents a potential therapeutic strategy, which requires precise differentiation of application scenarios. For diseases characterized by chronic inflammation and progressive damage (e.g., IIM, sarcopenia), inhibiting pro-inflammatory pyroptosis may be the primary approach. Potential strategies include targeting the NLRP3 inflammasome, blocking GSDMD/GSDME cleavage or pore formation (e.g., GSDME inhibition in sarcopenia models [159]), or neutralizing key pro-inflammatory cytokines such as IL-1β and IL-18. Conversely, in scenarios requiring enhanced regenerative capacity, such as aging-associated regeneration defects, leveraging or enhancing the pro-repair functions of pyroptosis is more promising. Based on the discovery of the GSDMD-11,12-EET axis, strategies such as elevation of 11,12-EET levels (e.g., inhibiting its degrading enzyme EPHX2 or supplementing analogs) or optimizing FGF signaling provide new avenues for promoting muscle regeneration [161]. Targeting the GSDME-IL-18-TRM axis is also promising [164]. The key is to use IL-18 to expand reparative tissue-resident macrophages to inhibit steatosis and reshape the regenerative microenvironment, which provides a new strategy for improving skeletal muscle repair. In conclusion, modulating pyroptosis can reduce inflammatory responses and improve muscle function in affected individuals.

### 3.6. The Role and Mechanism of Ferroptosis in Skeletal Muscle Diseases

**Figure 5:**
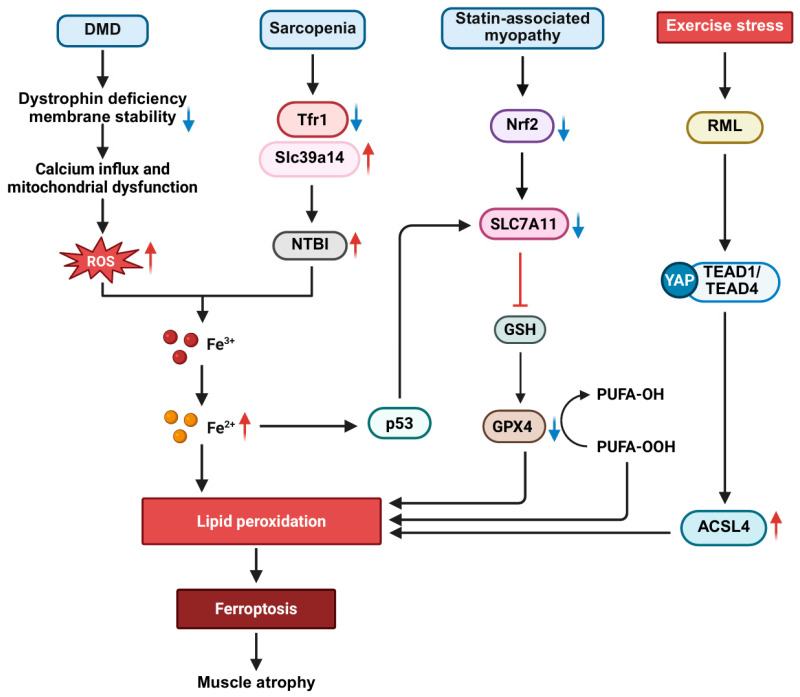
**Ferroptosis in skeletal muscle diseases.** In Duchenne muscular dystrophy (DMD), dystrophin deficiency leads to sarcolemmal instability, causing calcium influx and mitochondrial dysfunction. This results in excessive reactive oxygen species (ROS) production, which disrupts iron storage proteins (e.g., ferritin), releasing free iron (Fe^3+^/Fe^2+^). The free iron catalyzes the Fenton reaction, generating toxic hydroxyl radicals that initiate lipid peroxidation. In sarcopenia, aging-associated dysregulation of iron transporters, characterized by decreased TfR1 and increased ZIP14 (Slc39a14) expression, promotes the uptake of non-transferrin-bound iron (NTBI). This leads to intracellular iron overload, which drives lipid peroxidation and activates the p53-SLC7A11 axis, further depleting glutathione (GSH) and suppressing GPX4 activity. In statin-related myopathy, statins can directly induce ferroptosis in skeletal muscle cells by inhibiting the key intracellular antioxidant axis Nrf2-xCT/GPx4. In rhabdomyolysis (RML), exertional stress (e.g., heat stroke) activates the Hippo-YAP pathway, leading to YAP nuclear translocation and subsequent transcriptional upregulation of ACSL4. ACSL4 promotes the esterification of polyunsaturated fatty acids (PUFAs) into membrane phospholipids, increasing substrates for peroxidation. These disease-specific pathways ultimately converge on a common core executive mechanism. This mechanism involves the depletion of GSH (substrate for GPX4), accompanied by iron overload and ACSL4-mediated lipid remodeling, which together lead to uncontrolled peroxidation of polyunsaturated fatty acids, resulting in the generation of toxic lipid peroxides (PUFA-OOH). This lipid peroxidation cascade, unchecked by the compromised antioxidant systems, culminates in ferroptosis and subsequent muscle atrophy. Black arrows indicate promotion or activation. Blunt-end red arrows indicate inhibition. Red arrows indicate upregulation. Blue arrows indicate downregulation. Figure created with BioRender.com (https://biorender.com/q5c5uri).

Ferroptosis is an iron-dependent, non-apoptotic cell death modality characterized by abnormal accumulation of reactive ROS, smaller mitochondria, reduced mitochondrial cristogenesis, increased mitochondrial membrane density, and increased mitochondrial membrane disruption [14,165]. At present, the main known defense systems include the glutathione peroxidase 4 (GPX4) pathway and ferroptosis suppressor protein 1 (FSP1) pathway, as well as the dihydroxycombinol dehydrogenase (DHODH) pathway located in the mitochondria. Among them, GPX4 and FSP1 are the core pathways that have been most extensively studied at present. The core process of ferroptosis is the disorder of iron metabolism, which leads to the excessive production of reactive oxygen species and the failure of key antioxidant defense systems, such as GPX4 system, and eventually fatal lipid peroxidation. This form of cell death significantly contributes to sarcopenia, DMD, statin-induced myopathy, and RML (Figure 5).

In sarcopenia, age-related dysregulation of iron homeostasis may lead to tissue iron overload. Sarcopenia models indicate that this iron overload is associated with the activation of the ferroptosis pathway [166]. The potential mechanism may involve iron overload-mediated activation of the p53 protein, which suppresses the expression of the cystine transporter SLC7A11, reducing GSH synthesis and impairing GPX4 activity. This triggers lipid peroxidation and muscle cell death, ultimately resulting in muscle atrophy [166]. In addition, the accumulation of ions in skeletal muscle increases in aging or disease states, which generates a large number of ROS through the Fenton reaction, destroys the lipid structure of cell membrane, and induces ferroptosis. For example, in the skeletal muscles of naturally aging mice, the expression of transferrin receptor 1 (Tfr1) decreases with age, while the expression of Slc39a14 (encoding zinc transporter ZIP14) increases compensatorily. Although Slc39a14 is a zinc transporter, it can mediate the absorption of non-transferrin-bound iron (NTBI), leading to the accumulation of iron ions in the muscles and activating the ferroptosis pathway. This mechanism has been verified in an artificial Tfr1 knockout model (specific knockout of Tfr1 in satellite cells) [167].

In the context of DMD, iron accumulation and ferroptosis form a mutually reinforcing vicious cycle that significantly exacerbates muscle degeneration. For instance, studies using laser ablation inductively coupled plasma mass spectrometry (LA-ICP-MS) have demonstrated substantial iron overload in the hindlimbs of severely dystrophin/utrophin double knockout mice and in the diaphragm of moderately dystrophin-deficient mdx mice [168]. It is characterized by elevated total iron levels and increased expression of iron homeostasis-related proteins such as ferritin and transferrin [168]. This iron accumulation may be related to decreased membrane stability triggered by the loss of dystrophin. Dystrophin is an important muscle cytoskeleton protein. Dystrophin deficiency in DMD has been shown to disrupt the integrity of the sarcolemma [169,170], leading to mitochondrial dysfunction and oxidative stress [171], which in turn increases intracellular reactive ROS and peroxide levels. Ferritin is the main intracellular iron storage protein, and its structural stability depends on the normal cellular environment. When the integrity of the sarcolemma is lost, oxidative stress increases, possibly leading to structural disruption of ferritin and release of free iron ions. At the same time, the loss of membrane integrity may also affect the function of ferroportin (such as FPN1) located on the cell membrane, further hindering iron efflux. These factors may together form the basis of intracellular iron accumulation. It is worth noting that the coexistence of a free iron pool and a high level of peroxide in this pathological environment will cause the Fenton reaction, which produces hydroxyl radicals with high cytotoxicity [172]. These radicals cause extensive oxidative damage to lipids, proteins, and DNA [173], and an increase in oxidative stress levels is already a critical factor in the progression of muscle degeneration in DMD. Notably, the iron chelator deferiprone has shown therapeutic potential in the mdx mouse model by effectively reducing muscle iron levels, mitigating oxidative stress markers, and alleviating fibrosis [168]. However, while an iron-rich diet intervention increased iron storage proteins and some heme-containing proteins, it did not exacerbate the pathological features of muscular dystrophy [168]. This highlights that the core of muscle damage in DMD lies in the oxidative toxicity of iron rather than merely its content. Therefore, targeting ferroptosis pathways (e.g., regulating iron homeostasis and enhancing antioxidant capacity) is a promising new direction to preserve muscle function and delay atrophy in DMD.

The role of ferroptosis in statin-induced myopathy and RML has become increasingly clear. Evidence shows that statins (e.g., atorvastatin) can induce ferroptosis in skeletal muscle cells by inhibiting the critical intracellular antioxidant axis Nrf2-xCT/GPx4, thereby causing muscle-related symptoms such as weakness, pain, cramps, and even severe RML [174]. Furthermore, RML is also a major type of muscle injury associated with exertional heat stroke (EHS) [175,176]. In EHS models, the ferroptosis-specific inhibitor Ferrostatin-1 significantly improved the survival rate of EHS model mice and effectively suppressed the progression of EHS-induced RML [177]. The key executor of this pathological process, acyl-CoA synthetase long-chain family member 4 (ACSL4), is significantly up-regulated in EHS stimulation, which promotes skeletal muscle cell ferroptosis and subsequent RML by promoting muscle lipid peroxidation. Inhibiting ACSL4 markedly reduced EHS-induced ferroptosis in skeletal muscle cells, as indicated by reduced serum CK and myoglobin (MB) levels, and decreased the accumulation of lipid peroxidation products in the muscle [177]. Further studies showed that the Yes-associated protein (YAP) mediated the EHS-induced upregulation of ACSL4 by binding to the transcription factors TEAD1/TEAD4 [177]. Inhibition of YAP or ACSL4 or clearance of lipid peroxidation could prevent EHS-induced ferroptosis and ameliorate skeletal muscle tissue damage [177]. These findings reveal the important role of the ACSL4-YAP-TEAD regulatory axis in RML pathology and provide a new strategy for targeting ACSL4 to prevent and treat RML.

Ferroptosis is also associated with acute kidney injury (AKI) in RML. AKI is a common complication of RML. In RML-associated AKI, a large amount of myoglobin is released, and heme iron is lipid-soluble. The impaired cellular energy metabolism caused by iron deficiency, such as insufficient production of NADPH, limits the activity of Heme oxygenase 1 (HO-1), resulting in the inability of heme to be effectively degraded and its accumulation in the epithelial cells of the proximal tubules of the kidney. These free heme iron intercalates into the lipid layer of the cell membrane, induces significant lipid peroxidation, manifested as increased 4-HNE, and promotes cellular senescence by activating the p53/p21 pathway, ultimately exacerbating RML-induced AKI [178]. In addition, curcumin, as a powerful antioxidant, can inhibit ferroptosis, thereby improving RML-related AKI [179], which opens new avenues for the treatment of RML syndrome. These results further support the widespread effect of ferroptosis in muscle damage and related systemic complications.

In conclusion, ferroptosis is a central pathway connecting iron metabolism disorders, oxidative stress, and skeletal muscle cell death, playing a critical role in various myopathies. In sarcopenia, iron homeostasis disorders induce muscle atrophy by activating the p53-SLC7A11 pathway to inhibit GSH synthesis and weaken GPX4 activity. In DMD, the damage of membrane integrity, mitochondrial dysfunction and oxidative stress caused by dystrophin deficiency may lead to abnormal iron accumulation. Free iron and peroxide produce hydroxyl radicals through the Fenton reaction, forming a vicious cycle of oxidative damage and muscle degeneration. In statin-induced myopathy and RML, drugs or heat stress significantly promote skeletal muscle lipid peroxidation and ferroptosis by inhibiting the Nrf2-xCT/GPx4 pathway or activating the ACSL4-YAP-TEAD regulatory axis. In addition, myoglobin heme iron released by RML can be embedded in the lipid layer of renal cell membrane and aggravate ferroptosis-related AKI. Notably, interventions such as iron chelators (e.g., deferiprone), ferroptosis inhibitors (e.g., Ferrostatin-1), ACSL4 inhibitors, and curcumin have shown therapeutic potential in muscle disease models. These results not only confirm the critical role of ferroptosis in multiple muscle diseases but also highlight the value of targeting key nodes of ferroptosis as prevention and treatment of skeletal muscle diseases and related systemic complications.

### 3.7. The Role and Mechanism of Cuproptosis in Skeletal Muscle Diseases

Cuproptosis is a recently identified form of regulated cell death, first proposed by Peter Tsvetkov and colleagues in 2022 [180], which is distinct from other RCD mechanisms. It is triggered by the abnormal intracellular accumulation of copper and is cooperatively driven by ferredoxin 1 (FDX1) and lipoic acid synthetase (LIAS). LIAS catalyzes the lipoylation of key tricarboxylic acid (TCA) cycle enzymes (e.g., DLAT), creating specific binding sites for copper ions. Meanwhile, FDX1 reduces Cu^2+^ to the more reactive Cu^+^, which directly targets these lipoylated proteins, inducing their toxic oligomerization and the formation of insoluble aggregates [180]. In parallel, Cu^+^ competitively inhibits the biosynthesis of iron–sulfur (Fe-S) cluster proteins, leading to their destabilization and further exacerbating mitochondrial dysfunction.

**Figure 6:**
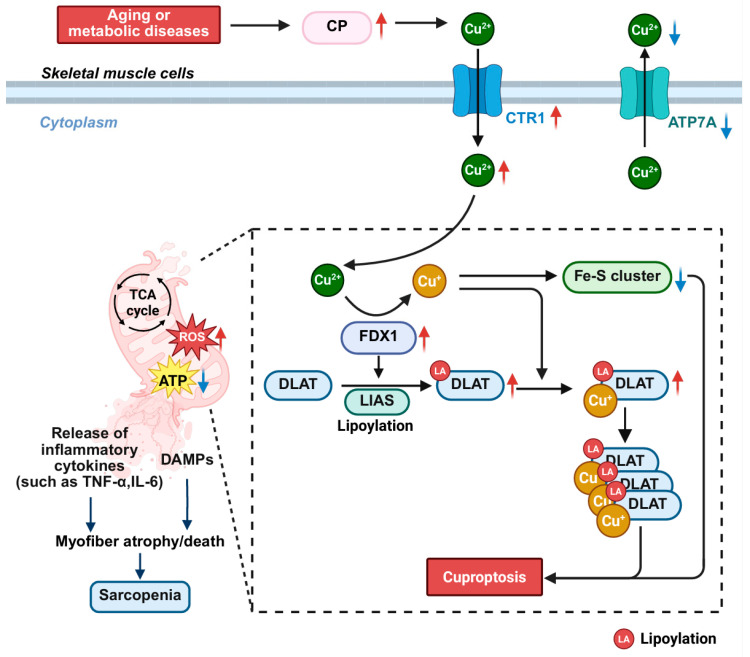
**Cuproptosis in skeletal muscle diseases.** In aging or metabolic disorders, elevated serum ceruloplasmin (CP) levels lead to increased circulating copper load. This extracellular copper overload promotes intracellular accumulation via upregulated copper importer CTR1 and/or dysfunctional copper exporter ATP7A within skeletal muscle cells. Accumulated Cu^2+^ is transported into mitochondria and reduced to the more reactive Cu^+^ by FDX1. Concurrently, FDX1 and the lipoic acid pathway activate the lipoylation of TCA cycle enzymes such as dihydrolipoamide S-acetyltransferase (DLAT). Cu^+^ directly binds to lipoylated proteins (e.g., DLAT), inducing their toxic oligomerization and aggregation, which disrupts the TCA cycle. Also, Cu^+^ leads to the destabilization of Fe-S cluster proteins. These processes together trigger severe mitochondrial dysfunction characterized by ATP depletion and ROS burst, ultimately leading to copper death. The collapse of mitochondrial function and integrity triggers inflammatory signaling and the release of DAMPs, which contribute directly to myofiber atrophy and loss of muscle mass and strength, hallmarks of sarcopenia. Black arrows indicate promotion or activation. Red arrows indicate upregulation. Blue arrows indicate downregulation. Figure created with BioRender.com (https://biorender.com/q5c5uri).

Cuproptosis, as a copper-dependent form of cell death, may exacerbate muscle atrophy by disrupting the mitochondrial respiration and energy metabolism processes, thereby contributing to the occurrence of sarcopenia (Figure 6). Studies have shown that in the mouse model of sarcopenia, the key driver gene FDX1 (which is responsible for reducing Cu^2+^ to the toxic Cu^+^) is significantly upregulated, directly promoting copper ion-dependent lipidated protein aggregation and mitochondrial membrane damage [181]. Meanwhile, the expression of multiple cuproptosis-related genes involved in energy metabolism (such as *PDHA1*, *DLAT*, *PDHB*, *NDUFC1*) was significantly decreased [181]. The genes such as *PDHA1* and *DLAT* are components of the pyruvate dehydrogenase complex (PDC), which is responsible for energy production. Their downregulation leads to the disruption of the TCA cycle, depletion of ATP, and imbalance of NADH/NAD^+^, thereby increasing mitochondrial ROS accumulation and copper ion retention [181]. Furthermore, the expression of copper uptake proteins (such as CTR1) in aged muscles increases, while the function of copper-excreting proteins (such as ATP7A) declines. This further promotes the accumulation of copper ions within muscle cells [182], ultimately leading to muscle fiber atrophy and loss of muscle function.

Furthermore, aging or metabolic diseases (such as MAFLD) can exacerbate copper metabolism disorders. Serum ceruloplasmin (CP)—which serves as the main copper source for tissues outside the liver, such as skeletal muscle—shows a significant increase in its circulating level under conditions of metabolic disorders (such as obesity and insulin resistance), resulting in extracellular copper overload characterized by an increased copper level in the blood circulation [183]. This extracellular copper overload is a key factor driving intracellular copper overload. The copper ions bound to CP travel through the bloodstream to the skeletal muscle and enter the muscle cells through the reductase (such as FDX1) and transporter proteins (such as CTR1) on the cell membrane, resulting in intracellular copper accumulation [183]. The copper ions accumulated within muscle cells not only damage mitochondrial function through oxidative stress (such as increased ROS levels) but also cause abnormal aggregation of lipidated proteins (such as DLAT) in the TCA cycle, ultimately triggering cuproptosis in muscle cells. This process is closely related to the occurrence of sarcopenia.

On the other hand, the expression levels of genes related to cuproptosis (such as *PDHA1*, *DLAT*, *PDHB*, and *NDUFC1*) are correlated with the degree of immune cell infiltration (such as M0-type macrophages and resting NK cells). For example, the expression of these genes is negatively correlated with the infiltration degree of M0-type macrophages and resting NK cells, and positively correlated with that of CD8^+^ T cells [181]. This suggests that immune cells may play a role in the process of sarcopenia, in which cuproptosis may be involved.

### 3.8. The Role and Mechanism of Disulfidptosis in Skeletal Muscle Diseases

Disulfidptosis is caused by the excessive accumulation of cystine and glucose starvation, resulting from the disulfide stress [184]. Glucose starvation inhibits the pentose phosphate pathway, resulting in the depletion of the key reducing agent NADPH [185]. At the same time, the high expression of SLC7A11 prompts continuous excessive intake of cystine [185]. In the absence of NADPH, cystine cannot be reduced, causing abnormal accumulation of disulfides (such as cystine) within the cells and triggering disulfide stress [184]. This death is mainly characterized by abnormal cross-linking of the actin cytoskeleton and impaired cytoskeletal function [184]. As a newly discovered mode of cell death, disulfidptosis is still in its infancy in the mechanistic studies of MG.

**Figure 7:**
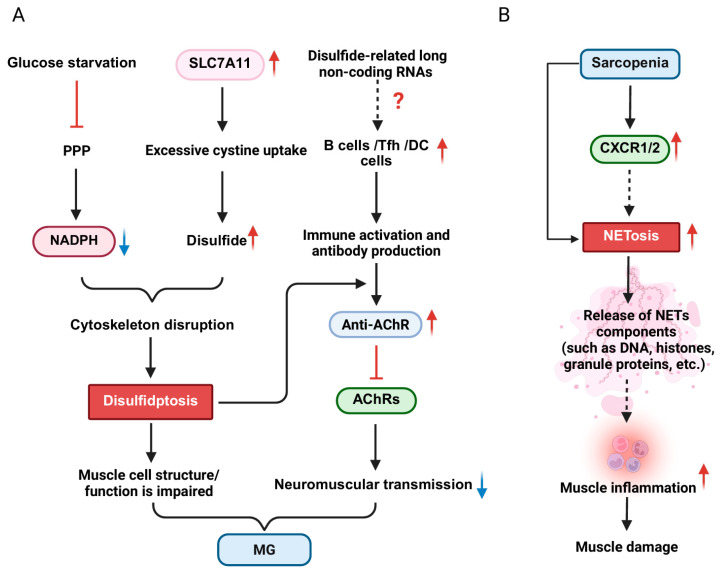
**Disulfidptosis and NETosis in skeletal muscle diseases.** (**A**) Glucose starvation inhibits the pentose phosphate pathway (PPP), leading to NADPH depletion, while high expression of SLC7A11 leads to excessive intake of cystine, which jointly triggers disulfide stress. Accumulated disulfides cross-link with cytoskeletal proteins, disrupting the network and executing cell death. In MG, this death pathway may promote the production of anti-achR autoantibodies by releasing autoantigens and remodeling the immune microenvironment (increasing B cells and T cells). Autoantibodies attack AChRs at the neuromuscular junction, impairing synaptic transmission and causing the clinical symptoms of MG. Also, in thymoma-associated MG, the expression signature of a set of disulfide-related long non-coding RNAs was identified, whose role and mechanism remain to be investigated. Thymoma patients with this characteristic have a significant increase in the infiltration of B cells, T follicular helper cells (Tfh) and dendritic cells (DCs) in the tumor immune microenvironment. Black arrows indicate promotion or activation. Blunt-end red arrows indicate inhibition. Red arrows indicate upregulation. Blue arrows indicate downregulation. (**B**) The mRNA expression levels of chemokine receptors *CXCR1* and *CXCR2* in peripheral blood cells of sarcopenia patients are increased, and the “NETs formation” pathway is enriched in the samples with high expression of *CXCR1/2*. In addition, the level of spontaneous formation of NETs by neutrophils was increased in the peripheral blood of sarcopenia patients. It is speculated that persistent activation of NETosis can induce muscle inflammation and subsequent muscle damage by releasing NET components (such as DNA, histones, granule proteins, etc.). However, the direct causal relationship between NETosis and sarcopenia needs to be further confirmed. Black arrows indicate promotion or activation. Red arrows indicate upregulation. Dashed arrows mean that the potential promotive effects need to be confirmed. Question marks (?) indicate unconfirmed or hypothetical regulatory relationships. Figure created with BioRender.com (https://biorender.com/q5c5uri).

Pan et al. first revealed its potential association with thymoma-related myasthenia gravis (TAMG) [186]. Based on the transcriptome data of thymoma patients in the TCGA database, this team identified 325 lncRNAs related to disulfidptosis. Among them, 25 were significantly differentially expressed between TAMG patients and non-TAMG patients (10 upregulated and 15 downregulated), and were co-expressed with classic disulfidptosis genes (*SLC7A11*, *SLC3A2*, *GYS1*, etc.) [186]. The study further constructed a prediction model composed of 11 disulfidptosis-related lncRNAs, which showed high prediction accuracy for TAMG risk (AUC = 0.934). In addition, the tumor immune microenvironment (TIME) of patients in the high-risk group based on this model showed unique immune cell infiltration characteristics, such as significantly increased infiltration levels of B cells, follicular helper T cells (Tfh) and dendritic cells (DCs) [186], suggesting that disulfidptosis-related pathways may not only directly damage the structure of muscle cells but also may promote the disease progression of MG by regulating the immune microenvironment, enhancing antigen presentation and promoting the production of autoantibodies (Figure 7A). Therefore, disulfidptosis-related genes may become an important driver and predictive marker for the initiation and progression of MG by disrupting cytoskeletal stability and reshaping the tumor immune microenvironment. However, this study mainly provides evidence of correlation at the transcriptome level, and the specific molecular mechanism of disulfidptosis in MG, whether disulfidptosis directly drives autoimmune responses, and its potential interaction with key antigens such as AChR need to be further studied.

### 3.9. The Role and Mechanism of NETosis in Skeletal Muscle Diseases

NETosis is a distinct form of inflammatory cell death in neutrophils, characterized by the release of neutrophil extracellular traps (NETs)—web-like structures composed of decondensed chromatin and granular proteins [187]. Upon sensing stimuli, neutrophils generate reactive ROS, which activate peptidylarginine deiminase 4 (PAD4). PAD4 catalyzes histone citrullination, leading to nuclear membrane rupture, chromatin decondensation, and its mixing with cytoplasmic enzymes such as elastase. The resulting NETs capture and kill pathogens [187]. However, excessive or dysregulated NETosis contributes to tissue damage and persistent inflammation, thereby exacerbating the progression of various diseases [188].

Recent studies have revealed the role of NETosis in the pathogenesis of sarcopenia, which has gradually attracted attention. Wang et al. integrated bioinformatics and machine learning to analyze the GSE226151 dataset and found that in the peripheral blood cell samples of sarcopenia patients, the mRNA expression levels of chemokine receptors *CXCR1* and *CXCR2* were increased [189]. Further pathway enrichment analysis (GSEA) suggested that in patient samples with higher *CXCR1/2* expression, the gene sets related to “formation of NETs” and “JAK-STAT signaling pathway” also showed higher expression levels [189]. In addition, Balazs et al. demonstrated through clinical studies that the spontaneous formation of NETs by neutrophils in peripheral blood was increased in sarcopenia patients. Because the NETs components (such as DNA, histones, granule proteins, etc.) released by NETosis have pro-inflammatory activity, and this release is in a state of continuous activation in sarcopenia, it is speculated that the enhancement of NETosis may lead to chronic muscle inflammation and subsequent muscle damage (Figure 7B). However, the direct causal relationship between NETosis and sarcopenia needs to be further confirmed.

## 4. Clinical Implications of Cell Death in Skeletal Muscle

### 4.1. Cell Death-Associated Biomarkers

Early diagnosis of skeletal muscle disorders relies on detecting specific molecular biomarkers associated with cell death modalities. CK, myoglobin, and pathway-specific markers—including Caspase-3 (apoptosis), gasdermin D (pyroptosis), and lipid peroxidation products (ferroptosis)—serve as potential biomarkers for assessing muscle injury and pathology. Novel biomarkers such as miR-434-3p demonstrate emerging diagnostic utility [68]. Real-time biomarker monitoring enables assessment of therapeutic response. For example, reduced Caspase activity following anti-apoptotic treatment or decreased gasdermin D cleavage fragments after pyroptosis inhibition indicates treatment efficacy. Notably, quantification of lipid peroxidation using C11-BODIPY 581/591 probes represents the gold-standard methodology for confirming ferroptosis [190]. In skeletal muscle diseases (such as sarcopenia and muscular dystrophy), ferroptosis-driven lipid peroxidation also leads to muscle cell atrophy and loss of muscle function. Measuring the level of lipid peroxidation in muscle tissue holds promise as a novel diagnostic indicator for muscle disorders.

### 4.2. Therapeutic Strategies Targeting Cell Death

Therapeutic approaches for skeletal muscle disorders increasingly focus on targeted modulation of cell death pathways. As a central pathological driver, multiple types of cell death orchestrate skeletal muscle injury progression. Systematically targeting these molecular pathways mitigates myofiber damage, delays disease advancement, and improves functional outcomes, constituting a major research frontier. Inhibition of necroptosis is applicable to inflammatory myopathies such as polymyositis. This pathway activates MLKL through RIPK1/RIPK3, forming necroptotic bodies that lead to cell lysis. The use of MLKL inhibitors (such as Necrosulfonamide) can effectively block this process, reducing muscle inflammation and fibrotic necrosis [113]. In addition, iron death inhibitors, such as Ferrostatin-1, are used to neutralize toxic lipid peroxides and protect muscle fibers. Studies have shown that in the skeletal muscles of aging mice, there is an iron metabolism disorder and iron death characteristics (such as decreased GPX4 activity). By intraperitoneal injection of Ferrostatin-1, iron death can be effectively inhibited, and the muscle quality and exercise endurance of elderly mice can be significantly improved (for example, better performance in the rod-wheel fatigue test) [167]. Moreover, by regulating the balance of Bcl-2 family proteins (Bax/Bcl-2) or inhibiting Caspase activity, apoptosis can be prevented. A study on muscle atrophy indicates that the drug losartan can significantly reduce muscle cell apoptosis by regulating the ratio of Bcl-2/Bax, thereby delaying the process of muscle atrophy [191].

Regulating the autophagy flux is crucial for age-related muscle atrophy. Autophagy is a core process for maintaining cellular homeostasis. On one hand, activating AMPK (such as through exercise) can phosphorylate Ulk1 to initiate autophagy and remove damaged mitochondria [130]. On the other hand, inhibiting mTORC1 (such as using rapamycin) can relieve its inhibition on autophagy [148]. Both of which have been proven to improve muscle mass and function. Additionally, some studies suggest that curcumin reduces lipid peroxidation through the Nrf2/HO-1 pathway, thereby inhibiting ferroptosis [179] and indirectly protecting muscles from damage related to RML.

The interaction of different cell death pathways is prevalent in skeletal muscle diseases. For example, inflammatory factors released by pyroptosis can exacerbate oxidative stress associated with ferroptosis [121]. Research indicates that overexpression of nuclear factor erythroid 2-related factor 2 (Nrf2) or knockdown of Kelch-like ECH-associated protein 1 (KEAP1) upregulates the expression of HO-1 (an iron-degrading enzyme) and GPX4, while promoting GSH synthesis. These effects collectively inhibit the ferroptosis pathway [192]. Moreover, it is important to note that when Nrf2 is effectively activated to reduce intracellular reactive ROS levels, it can suppress the pyroptosis pathway [193]. Disulfidptosis is closely related to ferroptosis. SLC7A11, as a specific cystine transporter, is a key protein in the regulation of ferroptosis and disulfidptosis. The down-regulation of SLC7A11 indirectly inhibits the activity of GPX4 by inhibiting the cysteine metabolic pathway, leading to a decrease in the intracellular cysteine level and the depletion of GSH biosynthesis, leading to the accumulation of lipid peroxides and finally inducing ferroptosis. At the same time, however, this process inhibits the occurrence of disulfidptosis [185,194]. Furthermore, the cross-regulation between NETosis and ferroptosis is also worthy of attention. Studies have shown that the lipid peroxides accumulated during ferroptosis can activate neutrophils and promote the formation of NETs [195]. The large amount of ROS released by NETs and the oxidative substances contained therein can further exacerbate lipid peroxidation and ferroptosis in muscle cells [196], forming a positive feedback loop.

Overall, these strategies are based on precise targets such as RIP1, AMPK and mTORC1, and offer therapeutic potential for various skeletal muscle diseases such as polymyositis and age-related myopathy. Nevertheless, the intricate interplay among these cell death modalities poses a fundamental challenge to single-agent pharmacotherapy. In the chronically stressed microenvironment of diseased skeletal muscle, apoptosis, necroptosis, and ferroptosis do not function as isolated on/off switches but rather constitute a redundant and interconnected biological circuit. Blockade of one specific lethal pathway may trigger compensatory activation of alternative death subroutines, thereby limiting long-term therapeutic efficacy. For example, in inflammatory myopathies, if Caspase-8 activity is inhibited by the pathological microenvironment or pharmacological intervention, the death signal switches from the classical apoptotic pathway to RIPK1/RIPK3/MLKL-mediated necroptosis, making it difficult to effectively prevent muscle fiber loss with Caspase 8-only inhibition therapy [113]. Consequently, future pharmacotherapeutic strategies for skeletal muscle pathologies must transition toward a multi-target paradigm or polytherapy approach to robustly preserve muscle function and achieve durable clinical outcomes.

## 5. Conclusions

Recent years have witnessed breakthrough advances in understanding cell death pathways in skeletal muscle disorders. Studies have confirmed that programmed death forms—such as apoptosis, necroptosis, autophagy-related cell death, pyroptosis, and ferroptosis—participate in the process of skeletal muscle injuries through different molecular pathways. Among these, necroptosis, mediated by the RIPK1/RIPK3/MLKL pathway, acts as a key pro-inflammatory driver, aggravating myofiber death and inflammatory damage in diseases like DMD and PM. The regulatory role of autophagy is particularly prominent in skeletal muscle disorders. Impaired autophagy can accelerate myofiber atrophy, while moderate activation of autophagy facilitates the clearance of abnormal protein aggregates to alleviate skeletal muscle disorders. Pyroptosis also exhibits a dual role in skeletal muscle diseases, encompassing pro-inflammatory destruction and pro-repair/regenerative effects. Ferroptosis disrupts sarcolemmal integrity via lipid peroxidation, showing a positive correlation with disease severity in DMD models.

Synthesizing current research, cell death in skeletal muscle diseases is characterized by the coexistence and interaction of multiple pathways. Classical RCD (such as apoptosis, necroptosis, autophagy-related cell death, pyroptosis, and ferroptosis) has been confirmed to be deeply involved in the occurrence and development of skeletal muscle diseases. Meanwhile, the roles of emerging death modalities (such as Cuproptosis, Disulfidptosis, NETosis) in skeletal muscle diseases are being progressively elucidated. A deep understanding of the unique mechanisms, mutual regulation, and dynamic changes in these death pathways in specific skeletal muscle pathological environments will not only help to elucidate the nature of the disease but also provide promising new directions for the development of targeted intervention strategies (such as specific pathway inhibitors, agonists, or metabolite modulators).

In summary, this paper systematically describes the important roles of multiple cell death, such as apoptosis, necroptosis, autophagy-related cell death, pyroptosis, ferroptosis, cupreptosis, and disulfidptosis, in a variety of skeletal muscle diseases. A deeper understanding of these complex mechanisms not only advances our knowledge of muscle disease pathophysiology but also provides essential guidance for refining therapeutic strategies. Future therapeutic breakthroughs are likely to rely on multi-target or combined interventions, with the core objective of more effectively preserving muscle structure and function, thereby improving clinical outcomes for patients.

## Data Availability

No new data was generated during this study.

## Abbreviations

The following abbreviations are used in this manuscript:

ACD	Accidental necrosis
AChR	Acetylcholine receptor
AChR-Ab	Acetylcholine receptor antibody
ACP	Acid Phosphatase
ACSL4	Acyl-CoA synthetase long-chain family member 4
AKI	Acute kidney injury
AMD	Acid maltase deficiency
ANT	Adenine nucleotide translocator
ATG	Autophagy-related
CK	Creatine kinase
CoQ10	Coenzyme Q10
CoQH_2_	Ubiquinol
CP	Ceruloplasmin
DAMPs	Damage-associated molecular patterns
DCs	Dendritic cells
DHODH	Dihydroxycombinol dehydrogenase
DISC	Death-inducing signaling complex
DLD	Dihydrolipoamide dehydrogenase
DMD	Duchenne muscular dystrophy
EHS	Exertional heat stroke
eIF5A1	Eukaryotic translation initiation factor 5A1
FAPs	Fibro-adipogenic progenitors
Fe-S	Iron–sulfur cluster
FGF2	Fibroblast growth factor 2
FSP1	Ferroptosis suppressor protein 1
GAA	Acid alpha-glucosidase
GPX4	Glutathione peroxidase 4
GSD	Glycogen storage diseases
GSDMD	Gasdermin D
GSDME	Gasdermin E
GSH	Glutathione
HMGB1	High-mobility group box 1 protein
HO-1	Heme oxygenase 1
IAPs	Inhibitors of apoptosis proteins
IBM	Inclusion body myositis
IHSMC	Immortalized skeletal muscle cells
IIM	Idiopathic inflammatory myopathy
IL-1β	Interleukin-1β
IMM	Inner Mitochondrial Membrane
IOPD	Infantile-onset Pompe disease
KEAP1	Kelch-like ECH-associated protein 1
LA-ICP-MS	Laser ablation inductively coupled plasma mass spectrometry
LIAS	Lipoic acid synthetase
LIPT1	Lipoyltransferase 1
LLPS	Liquid–liquid phase separation
lncRNAs	Long non-coding RNAs
LOPD	Late-onset Pompe disease
LPS	Lipopolysaccharide
LRP4-Ab	Low-density lipoprotein receptor-related protein 4 antibody
MELAS	Mitochondrial encephalopathy, lactic acidosis and stroke-like episodes
MG	Myasthenia gravis
MLKL	Mixed lineage kinase domain-like protein
MM	Mitochondrial myopathy
MOMP	Mitochondrial outer membrane permeability
mPTP	Membrane permeability transition pore
mtDNA	Mitochondrial DNA
MuSCs	Muscle stem cells
MuSK-Ab	Muscle-specific receptor tyrosine kinase antibody
nDNA	Nuclear DNA
NETs	Neutrophil extracellular traps
Ninj1	Nerve Injury-Induced Protein 1
NMJ	Neuromuscular junction
Nrf2	Nuclear factor erythroid 2-related factor 2
NTBI	Non-transferrin-bound iron
OMM	Mitochondrial Outer Membrane
OXA1L	Oxidase Assembly 1-like protein
OXPHOS	Oxidative phosphorylation
PAMPs	Pathogen-associated molecular patterns
PAD4	Peptidylarginine deiminase 4
PBMCs	Peripheral blood mononuclear cells
PDC	Pyruvate dehydrogenase complex
PM	Polymyositis
P-S65-Ubiquitin	Phosphorylated Ubiquitin at Ser65
PTX3	Pentraxin 3
PUFA-PL-OHs	Phospholipid alcohols
PUFA-PL-OOHs	Phospholipid hydroperoxides
PUFA-PLs	Polyunsaturated fatty acid-containing phospholipids
PUMA	P53-upregulated modulator of apoptosis
RCD	Regulated cell death
RIPK1	Receptor-Interacting Protein Kinase 1
RIRR	ROS-induced ROS release
RML	Rhabdomyolysis
ROS	Reactive oxygen species
RRFs	Ragged red fibers
SIRS	Systemic inflammatory response syndrome
SMAC	Second Mitochondria-derived Activator of Caspases
TAMG	Thymoma-related myasthenia gravis
Tfh	Follicular helper T cells
Tfr1	Transferrin receptor 1
TIME	Tumor immune microenvironment
Tregs	Regulatory T cells
TRMs	Tissue-resident macrophages
ULK1	Unc-51-like autophagy activating kinase 1
UPR	Unfolded protein response
UPS	Ubiquitin–proteasome system
VDAC	Voltage-dependent anion channel
XIAP	X-linked inhibitor of apoptosis protein
YAP	Yes-associated protein
